# Human amylin is a potent antimicrobial peptide that exhibits antimicrobial synergism with the amyloid beta protein

**DOI:** 10.1002/alz.70490

**Published:** 2025-07-29

**Authors:** Deepak K. Vijaya Kumar, Teryn A. Mitchell, Breeya A. Tailor, Alexander P. Moir, Nanda K. Navalpur Shanmugam, William A. Eimer, Jessica Ghelichi, Se Hoon Choi, Chienwen Su, Alex S. Rodriguez, Eunhee Kim, Robert D. Moir, Rudolph E. Tanzi

**Affiliations:** ^1^ Genetics and Aging Research Unit Henry and Allison McCance Center for Brain Health MassGeneral Institute for Neurodegenerative Disease Department of Neurology Massachusetts General Hospital and Harvard Medical School Charlestown Massachusetts USA; ^2^ Mucosal Immunology and Biology Research Center Massachusetts General Hospital and Harvard Medical School Charlestown Massachusetts USA; ^3^ Present address: Department of Physiology Korea University College of Medicine Seoul Republic of Korea

**Keywords:** Alzheimer's disease, amylin, amyloid beta, antimicrobials, bacteria, diabetes, fungi, host, immunity, infection

## Abstract

**INTRODUCTION:**

Our previous studies demonstrated the antimicrobial properties of amyloid beta (Aβ) of Alzheimer's disease (AD) against clinically relevant bacteria, yeast, and viruses. In this study, we investigate the antimicrobial function of the 37‐amino acid amylin of type 2 diabetes (T2D), expanding on its potential involvement in AD.

**METHODS:**

We used in vitro assays, including human three‐dimensional neuronal cell culture models, to test microbicidal, microbiostatic, and synergistic antimicrobial interactions between amylin and Aβ against microbes.

**RESULTS:**

Our results confirm that amylin is a broad‐spectrum antimicrobial peptide that exhibits both microbicidal and microbiostatic mechanisms. We also identified a synergistic antimicrobial effect between amylin and Aβ in inhibiting *Salmonella* Typhimurium and *Staphylococcus aureus*.

**DISCUSSION:**

The findings show that amylin is an antimicrobial peptide and functions synergistically with Aβ against bacterial pathogens. Increased amylin secretion after bacterial infection suggests a broader biological role for amylin beyond its involvement in T2D.

**Highlights:**

- Amylin is a potent antimicrobial peptide, eliminating ≥99.9% bacteria at low doses.
- Amylin efficiently traps and neutralizes microbes via a fibril‐driven mechanism.
- Amylin protects human cells and *Caenorhabditis elegans* from *Salmonella* or *Candida* infection.
- Synthetic amylin and amyloid beta (Aβ) together amplify antibacterial response against bacteria.
- Synergy between cell‐derived amylin and Aβ drives dynamic antimicrobial activity against neural infection.

## 1 BACKGROUND

Alzheimer's disease (AD) and diabetes are among the top 10 leading causes of death in the United States, making them two of the most critical age‐related diseases.^1^, ^2^ Over 90% of all diagnosed diabetes cases are type 2 diabetes (T2D).^3^ Despite distinct clinical manifestations, AD and T2D exhibit many similar pathological hallmarks, including chronic inflammation, cell death, metabolic dysfunction, and, perhaps most strikingly, protein aggregation. AD neuropathology is primarily characterized by the accumulation of amyloid beta (Aβ) plaques and intracellular neurofibrillary tangles (NFTs) composed of hyperphosphorylated tau protein,^4^ whereas T2D is marked by pancreatic amyloid plaques composed of the protein amylin, also known as islet amyloid polypeptide (IAPP).^5^, ^6^, ^7^, ^8^, ^9^ The pathological mechanisms involved in the respective proteinopathies of AD and T2D show remarkable parallels, particularly concerning Aβ and amylin. Like the Aβ protein, amylin oligomerizes, generates reactive oxygen species (ROS), and aggregates as pancreatic plaques after undergoing tertiary conformational changes.^10^, ^11^ These aggregates disrupt cellular communication, induce inflammation, and cause cell death and tissue damage.^12^, ^13^

Despite their pathological roles, both proteins serve important physiological functions in healthy individuals. Amylin is co‐secreted with insulin by pancreatic β‐cells and regulates glucose homeostasis, satiety, and gastric emptying.^14^ Similarly, the amyloid precursor protein (APP), from which Aβ is derived, is involved in neuronal development and synaptic plasticity and also contributes to iron homeostasis. APP facilitates iron export from neurons through its ferroxidase‐like activity,^15^ and its expression is post‐transcriptionally regulated by an iron‐responsive element in the 5′ untranslated region of its mRNA, linking its production to systemic iron levels.^16^ These physiological roles suggest that Aβ and amylin may have evolved multifunctional roles, including contributions to innate immunity.

Contrary to its traditional classification as a harmful pathological byproduct, Aβ has been shown by our earlier work to be a natural antimicrobial peptide (AMP) against bacteria, fungi, and viruses.^17^, ^18^, ^19^ Although amylin's bacteriostatic activity against *Escherichia coli* and *Staphylococcus aureus* has been reported previously,^20^ its full range of antimicrobial spectrum, such as microbicidal and entrapment of target microbes, remained unexplored. Our study characterizes amylin's pronounced microbicidal activity and provides direct visual validation of a never‐before‐seen fibril‐mediated facet via microbe entrapment, confirming its identity as a bona fide amyloidogenic AMP.

Human AMPs can complex with each other functionally to enhance their pathogen‐targeting ability.^21^ Although antimicrobial synergy has been documented for many non‐amyloidogenic AMPs, investigating its occurrence and relevance to amyloidogenic AMPs remains underexplored.^22^ Amyloidogenic AMPs present the potential for additional synergistic activities not seen in non‐amyloidogenic AMPs^23^ because their tertiary conformational changes dictate much of their antimicrobial effects. Cross‐seeding of different amyloids can modify their aggregation dynamics, distinct from when they are alone,^23^ potentially producing novel antimicrobial effects.

Aβ and amylin have been detected independently and in co‐localized states in the cerebrospinal fluid (CSF) and brains of patients with AD and T2D.^24^, ^25^, ^26^ Cross‐seeding between Aβ and amylin has been suggested to be a catalyst for amyloid aggregation in AD and T2D brains.^27^, ^28^, ^29^, ^30^ However, the purpose of the cross‐seeding phenomenon between Aβ and amylin had remained undefined.^28^, ^29^, ^30^ Here, we demonstrate for the first time that amylin and Aβ cross‐seeding is a purpose‐driven design aimed at amplified antimicrobial response. This never‐before‐reported occurrence reveals a previously unrecognized synergy between two human amyloidogenic proteins aimed at bolstering host defense in the brain and pancreas.

Our study shows that not only is amylin a powerful stand‐alone AMP, but it synergizes with Aβ to mediate precise target‐specific pathogen elimination. These results reshape our understanding of amyloidogenic proteins as dual‐purpose agents that contribute to both host defense and, when dysregulated, disease pathogenesis. These findings have significant implications for delineating the physiological roles of amyloid peptides, particularly toward translational therapeutic advances for two of today's most significant age‐related diseases, AD and T2D. In addition, data support our hypothesis that pathological hallmarks of AD and T2D may stem from multifaceted AMP‐mediated immune responses initially aimed at protecting the brain and pancreas from microbial invasion.

## 2 METHODS

### 2.1 Synthetic peptides

Synthetic islet amyloid polypeptide (IAPP) or amylin was prepared and purified by Dr James I. Elliott at Yale University (New Haven, CT) using solid‐phase peptide synthesis. To disrupt aggregates generated during synthesis,^31^ bulk powdered peptides were dissolved in 30% trifluoroethanol (TFE) at 1 mg/mL, lyophilized (500 µL aliquots) in siliconized 1.7 mL sample tubes, and then resuspended at 1 mg/mL in 70% hexafluoroisopropanol (HFIP) and incubated at room temperature (RT) for 18 h. Following incubation, peptides were lyophilized a second time and stored under nitrogen at –20°C. Stock solutions at 1 to 1.5 mg/mL were prepared the day of experimentation by solubilizing peptide films in sterile water. Peptide concentrations in stock solutions were determined immediately before use by bicinchoninic acid (BCA) protein assay.

### 2.2 Bacterial and nematode strains

The following strains were purchased from American Type Culture Collection (ATCC): *Enterococcus faecalis* ATCC 29212, *Streptococcus agalactiae* ATCC 12386, *Candida albicans* ATCC 90028, *Listeria monocytogenes* ATCC 19112, *Staphylococcus aureus* ATCC 25923, and *Pseudomonas aeruginosa* ATCC. *Salmonella* Typhimurium SL1344 with streptomycin resistance (a kind gift from Dr Bobby Cherayil, MGH/Harvard Medical School, MA)

### 2.3 Antimicrobial assay by tube dilution method and spot tests

Serial two‐fold dilutions of amylin peptide with concentrations ranging from 100 µg/mL to 0.4 µg/mL were prepared in 5 mL polypropylene tubes. A 1:200 dilution of overnight Luria‐Bertani (LB) cultures of bacteria and yeast peptone dextrose (YPD) cultures of *Candida* were grown to mid‐log phase at 37^°^C and 80 rpm. The mid‐log phase cultures were added to each concentration of the amylin tubes, to obtain a 1 mL total volume of autoclaved distilled water containing appropriate amylin concentration combined with ≈2.5–5 × 10^5^ colony‐forming units (CFU)/mL bacteria or yeast. Control or amylin‐untreated tubes contained only 2.5–5 × 10^5^ CFU/mL bacteria or yeast in water. The tubes were incubated for 1 h in a 37^°^C incubator at 80 rpm. Immediately following 1 h incubation, the contents of the tubes, including those of the controls, were spot tested using 50 µL of undiluted suspensions on LB, blood agar (BA), or YPD agar, starting from 50 µg/mL. The remaining contents were serial‐diluted, and appropriate dilutions were spread‐plated on LB or YPD agar. The plates were incubated overnight in a 37°C incubator and the colonies counted the following day.

### 2.4 Antimicrobial assay using 96‐well polypropylene plate

The minimum inhibitory concentration (MIC) assay to determine the micro‐biostatic activity of amylin was carried out as per the classical microtitre broth dilution from the National Committee of Laboratory Safety and Standards (NCLSS). Sterile polypropylene 96‐well plates (Becton Dickinson ‐ BD Falcon) were used to test the susceptibility of bacterial and yeast species to amylin with a two‐fold dilution of peptide concentration from 100 µg/mL to 1.5 µg/mL. Control wells contained 100 µL Mueller Hinton Broth (MHB) with bacteria and 11 µL vehicle (0.01% acetic acid and 0.2% bovine serum albumin [BSA]). The assay was set up in quadruplicates for each pathogen with each well containing 11 µL of peptide and 100 µL of the respective bacterium in MHB(Cat. No. 212322, BD). The 96‐well plates were incubated at 37°C for 24 h, and the MIC was determined by the visual and microscopic inspection of wells that showed clumping at the lowest peptide concentration. Counts were obtained from two independent experiments per microorganism.

**RESEARCH IN CONTEXT**

1. **Systematic review**: The authors reviewed the published literature using PubMed and Google Scholar. Previous studies demonstrated the antimicrobial activities of amyloid beta (Aβ) and reported Aβ‐amylin co‐localization in Alzheimer's disease (AD) brain tissues. Some studies suggested that Aβ‐amylin cross‐seeding potentiates amyloid aggregation. The exact underlying cause of cross‐seeding remains unexplored.
2. **Interpretation**: Our results show that amylin is a potent antimicrobial peptide. When combined with Aβ, amylin enhanced antibacterial effects against *Salmonella* Typhimurium and *Staphylococcus aureus*, indicating a synergistic antimicrobial association between the two peptides. In human three‐dimensional cell infections, amylin and Aβ synergistically complexed to trap and sequester pathogens, suggesting that microbial pathogens may catalyze Aβ‐amylin cross‐seeding and co‐localized aggregation, thereby boosting innate immune response.
3. **Future directions**: Further research with more human pathogens in human cell culture and in vivo amylin and Aβ models will validate our findings. This would also further elucidate the potential early causes and implications of infection‐induced Aβ‐amylin cross‐seeding in the brain.

### 2.5 Time‐dependent antimicrobial assay by tube dilution method

Ten micrograms per milliliter of amylin peptide in water was prepared in triplicate in 5 mL polypropylene tubes. A 1:200 dilution of overnight LB cultures of bacteria or YPD cultures of *Candida* were grown to mid‐log phase at 37°C and 80 rpm. The mid‐log phase cultures were added to each of the amylin tubes, to obtain a 1 mL total volume of autoclaved distilled water containing amylin combined with ≈2.5–5 × 10^5^ CFU/mL bacteria or yeast. Control or amylin‐untreated tubes (triplicates) contained only ≈2.5–5 × 10^5^ CFU/mL bacteria or yeast in water. The tubes were incubated in a 37^°^C incubator at 80 rpm. The contents of the tubes were spot tested and plated following a 5 min, 15 min, and 30 min incubation on LB or YPD agar. The plates were incubated overnight in a 37°C incubator, and the colonies were counted the following day. Counts were obtained from three independent experiments per microorganism.

### 2.6 Bright field and fluorescence microscopic inspection of clumps

Ten microliters of broth from the test and control wells were gently smeared on clean and dry glass slides and allowed to air‐dry. Safranine dye was applied to the smear for 30 s and gently rinsed under tap water. The slides were examined under a bright field microscope at 4x. Ten microliters of broth from the test and control wells containing red‐fluorescent *S*. Typhimurium *SL1344* was gently smeared on clean and dry glass slides and air‐dried. The slides were stained with Thioflavin and mounted with Prolong Gold antifade reagent (Life Technologies) before viewing with a fluorescence confocal microscope (Leica TCS SL, Leica Microsystems, Germany).

### 2.7 Transmission electron microscopy

Clumps from amylin treated wells (5 µL) and 5 µL broth from control wells were absorbed to Formvar carbon‐coated copper grids (FCF100‐Cu, Electron Microscopy Sciences, Hatfield, PA, USA). Grids were blocked with 1% BSA in phosphate‐buffered saline (PBS; kept covered for 10 min at RT) and then incubated (30 min at RT) with Rb pAb to amylin (Abcam) 1:100 in blocking buffer. Grids were washed with PBS (x3) and incubated with goat anti‐rabbit‐IgG antibody covalently linked to nanogold particles (20 nm). Specimens were washed with water, stained with uranyl acetate, and then viewed using a JEM‐1011 Transmission Electron Microscope (JEOL Institute, Peabody, MA, USA).

### 2.8 Scanning Electron Microscopy

Glass coverslip surfaces were pretreated with 0.1% poly‐L‐lysine, and a drop of the sample (clumps from amylin‐bacteria/yeast aggregates) was added and allowed for attachment. This sample was placed in a fixative: 2.5% glutaraldehyde in 0.1 M NaCacodylate buffer pH 7.2. Samples were transported to the electron microscopy core facility at Northeastern University for post‐fixation in 1% osmium tetroxide. This was followed by dehydration through a graded ethanol series and critical point drying before imaging.

### 2.9 Host cell monolayer model

Host cell monolayers were prepared from human neuroglioma (H4) cell lines.^32^ H4 cells were maintained in Dulbecco's Modified Eagle Medium (DMEM) containing 10% fetal bovine serum (FBS), 2 mM L‐glutamine, 100 U penicillin, and 100 µg/mL streptomycin. Confluent cells were washed in PBS, trypsinized, and centrifuged. The pellet was washed again to remove any trace trypsin and antibiotics, and then resuspended in antibiotic‐free DMEM with 5% FBS and 2 mM L‐glutamine. Cell concentrations were determined by automated cell counter (TC20 BioRad, Hercules, CA, USA) and adjusted to 300,000 cells/mL. Host cell suspensions were transferred to the wells of six‐well culture plates and incubated for 48 h in 2 mL media/well. Cell confluence in plate wells was confirmed by microscopic examination.

### 2.10 *Assay of S*. Typhimurium invasion into H4 cells

*S*. Typhimurium (2.5–5 × 10^7^ CFU) was added to H4 cell monolayers, followed by 1 h incubation at 37°C. Monolayers were assayed for internalized bacterial populations with the gentamycin protection assay.^33^ Cell‐associated bacteria were killed by treating the monolayers with 100 µg/mL gentamycin for 90 min followed by three thorough PBS rinses. The internalized bacteria were released from the cells in 500 µL of 1% Triton X‐100 at 4°C. The 1% Triton X‐100–treated lysates were serial diluted and plated on LB agar plates and the CFU determined by colony count after an overnight incubation at 37°C.

### 2.11 *Caenorhabditis elegans* model

The wild‐type *C. elegans* N2 strain was used in our infection and rescue studies. Worms were synchronized as per established protocol, and the L1 larvae obtained were incubated on *E. coli* OP50 lawns for 48 h at 20°C to generate L4 larvae.^34^ L4 worms were incubated on *Candida albicans* lawns (2 h at 25°C), washed with M9 buffer to remove any surface *Candida* from the worms, and transferred to 35 mm polypropylene dishes containing 10 µg/mL amylin in M9 buffer or vehicle (M9 buffer). The *Candida*‐infected worms were incubated in the amylin solution/vehicle for 3 h at 25°C before being transferred to six‐well culture plates containing 1.5 mL/well of incubation media (79% M9 buffer, 20% brain heart infusion (BHI) broth, 10 µg/mL cholesterol, and 90 µg/mL kanamycin). The six‐well plates were incubated at 25°C overnight. The next day, the dead worms were removed and the survivors were treated again with 10 µg/mL amylin/vehicle for 3 h at 25°C followed by the six‐well transfer and overnight incubation in media. Nematodes were monitored the next day, and the surviving ones were transferred to fresh six‐well plates with the incubation media. The worms were monitored for 4 days after two rounds of amylin/vehicle treatment (3 h duration on Days 1 and 2). Hyphal penetration of the worm cuticle and death characterize *Candida*‐induced mortality in worms.

### 2.12 Human 3D ReN cell model, human primary astrocytes, and *Salmonella* infection

Human three‐dimensional (3D) neural (3D ReN) cells were derived from human neural progenitor cells (hNPCs) and cultured in 3D Matrigel. This environment mimics a brain‐like structure containing integral proteins such as laminin, entactin, and collagen. Furthermore, it allows us to recapitulate two hallmarks of the aged AD brain: plaque aggregation and tangle formation. To begin, a Matrigel stock was moved from the –80 to 4°C refrigerator 1 day before use. Then a 1:100 dilution of Matrigel (kept on ice) was made in cold DMEM/F12 medium. T75 flasks were coated with 8 mL of Matrigel solution and set for 1 h at RT before use. After 1 h media was aspirated and flasks were placed in 4C wrapped in Saran Wrap. Precoated flasks were used within 1 week.

Frozen stocks of ReN cells, G2B2 and A5, were thawed in a 37°C water bath and diluted in 6 mL of ReN expansion media. Next, the cells were centrifuged at 12,000 rpm for 5 min before resuspending in 10 mL of new media. This solution was transferred into pre‐coated flasks in a 37°C incubator until confluent ≈3 days after.

Once the cells reached confluency, media from each flask was aspirated and washed with 5 mL Dulbecco's‐PBS (D‐PBS). Two milliliters of warmed Accutase was added and placed into a 37°C incubator for 3–5 min until detachment. Four milliliters of pre‐warmed differentiation media was then added to each flask. Next, suspended cells were transferred to a sterile 15 mL conical vial and spun down at 12,000 rpm for 5 min. Cells were resuspended with 1 mL cold ReN differentiation medium and vortexed for 10 s. An aliquot of cells was extracted and diluted 1:10 for cell count. A 12‐well plate with 3 million cells per well was made with a cold differentiation medium.

In addition, the final volume of the cell mixture was diluted with a 1:11 Matrigel. The pipette tip was chilled with the cold medium before plating half with G2B2 and A5 and placed at 37°C overnight. The following day, the media was replenished. Media changes occurred 3 times a week until cells were differentiated at Week 3.

Primary human astrocytes were cultured according to the manufacturer's protocol using astrocyte growth medium (Gibco, N7805‐200, N7805‐100, and A1261301).

Freezer stocks of *S*. Typhimurium (SL1344) were thawed, and the colonies were maintained on LB agar with 100 µg/mL streptomycin and kept at 4°C. The bacterium was sub‐cultured every 2 weeks. The day before *Salmonella* infection, a single colony was suspended in 5 mL of LB with 100 µg/mL streptomycin broth and incubated overnight at 37°C. 1 mL *Salmonella* culture was centrifuged at 12,000 rpm at RT for 2 min. The supernatant was discarded, and the pellet was resuspended in D‐PBS (repeat). After two washes, *Salmonella* was reconstituted in 500 µL D‐PBS. Two tubes labeled A and B contained a 1:5 dilution of *Salmonella* in PBS. Tube A was heat‐killed, whereas tube B was not. Once cells were differentiated for 3 weeks, media was discarded and 1 mL of fresh media was added to each well before infection at different time points.

### 2.13 Human amylin enzyme‐linked immunosorbent assay (ELISA)

The Human amylin ELISA kit (Raybiotech, cat no. EIA‐AMY) was purchased to test the amylin levels of supernatants. All reagent preparation was followed as instructed. After adding samples, the plate was incubated overnight at 4°C with gentle shaking before washing and aliquoting the Streptavidin solution. The duration of the protocol was followed as instructed, and samples were read at 450 nm.

### 2.14 Double‐immunogold labeling (IGL) and transmission electron microscopy (TEM) imaging of *Salmonella* Typhimurium aggregate pellets after A5 and G2B2 cell infection

The Human amylin ELISA kit (Raybiotech, cat no. EIA‐AMY) was purchased to test the amylin levels of supernatants. All reagent preparation was followed as instructed. After adding samples, the plate was incubated overnight at 4°C with gentle shaking before washing and aliquoting the Streptavidin solution. The duration of the protocol was followed as instructed, and samples were read at 450 nm.

### 2.15 ELISA for detecting Aβ40, Aβ42, and Aβ38

The ELISA protocol was followed as per Meso Scale Discovery (MSD). Aβ42, Aβ40, and β38 calibrators were thawed and kept on ice. D35 (150 µL) was added to each well of a 96‐well plate and gently agitated on a shaker for 1 h at 100 rpm. Eight prepared standards were labeled tubes A–H. Ten microliters of Aβ38, 40, 42, and 370 µL of diff media were added to Tube A. To tubes B–H, 300 µL of diff media was added. Serial dilution (100 µL) was performed and was set aside on ice. The prepared detection antibody was labeled detection antibody. A total of 2940 µL of D100 and 60 µL of anti‐amyloid beta 6e10 was prepared. The plates were washed three times with 150 µL of wash buffer. Twenty‐five microliters of detection antibody and 25 µL of samples were added to each well. The plate was incubated overnight at 4°C with gentle rocking and shaken at 1000 rpm for 30 min at RT. The plates were washed three times with 150 µL wash buffer. Read buffer was prepared 1:1 with deionized (DI) water and 100 µL was added to each well, and read.

### 2.16 Immunohistochemistry (Astrocyte immunostaining)

Day 1: Free‐floating sections in 440 µm polyester mesh netwell (Costar) were washed with Tris‐Buffered Saline (TBS) for 10 min on a rocker, then washed twice with TBS+ (4% Triton X diluted in TBS) for 10 min each. With gentle rocking, the tissues were incubated with 5% Donkey serum diluted in TBS++ (4% Triton X) for 1 h at RT. Sections were probed overnight at 4°C in the dark with gentle rocking using a 1:500 dilution of anti‐amylin monoclonal rabbit (BMA biomedical). Day 2: Sections were washed with TBS three times at RT and incubated anti‐amylin 647 secondary antibody for 2 h at RT with gentle rocking. Sections were washed in 3 × 5‐min washes and stained with 1:10,000 probe of DAPI (Thermo Scientific) for 10 min, and then gently mounted on microscope slides (Fisher Scientific) with Prolong Gold anti‐fade reagent (Invitrogen) and imaged with confocal microscopy on a Nikon C2 laser scanning microscope.

#### 2.16.1 Histological co‐staining of post‐mortem human brain tissue

Formalin‐fixed, paraffin‐embedded post‐mortem brain sections from clinically confirmed AD patients (obtained from MGH Brain Biobank) were subjected to histological immunostaining. Co‐staining was performed using antibodies against human amylin (ab115766 and ab55411) and bacterial markers (Gram‐positive and Gram‐negative, ab20344 and ab41201). To ensure specificity, distinct amylin antibodies were used depending on the bacterial marker. Staining was visualized using standard chromogenic detection and imaged using brightfield microscopy.

#### 2.16.2 Statistical analysis

Data are expressed as mean values ± standard error of the mean (mean ± standard error of the mean). Error bars in the figures represent standard error of the mean. Statistical analyses were done by two‐tailed one‐sample *t* and Wilcoxon test (^****^ *p* < 0.0001), Mantel‐Cox and the Gehan‐Breslow‐Wilcoxon tests (^****^ *p* < 0.0001), and two‐way ANOVA and Sidak's multiple comparisons test.

## 3 RESULTS

### 3.1 Bactericidal and fungicidal activity of synthetic amylin against various clinical pathogens

**FIGURE 1:**
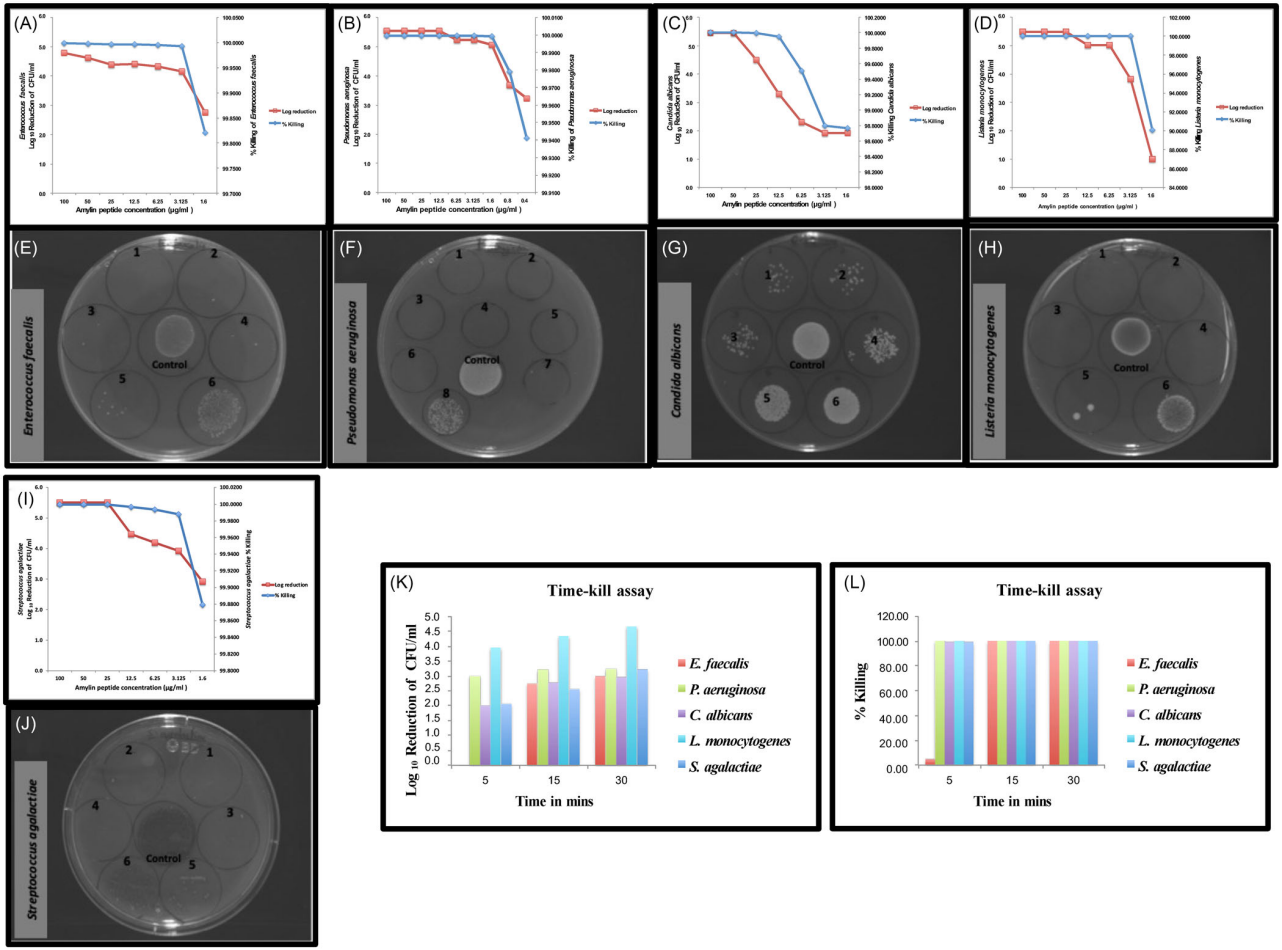
**Bactericidal and fungicidal activity of synthetic amylin against various clinical pathogens: (**A) *Enterococcus faecalis*, (B) *Pseudomonas aeruginosa*, (C) *Candida albicans, (D) Listeria monocytogenes*, and (*I). Streptococcus* *agalactiae*. **(A–D, I)** Dose‐dependent antimicrobial effects of cyclized amylin are expressed in log_10_ reduction of CFU/mL on the primary y‐axis. Percent reduction of bacteria/yeast corresponding to log_10_ reduction values with a 3‐log reduction ≥99.9% killing are correspondingly represented on the secondary y‐axis. Approximately 2.5–5 × 10^5^ CFU/mL of bacteria/yeast was used in the assay, and minimum bactericidal concentration (MBC) of amylin is the concentration that shows at least a 3‐log reduction (≥99.9% killing) of bacteria/yeast. CFU numbers were obtained from two independent experiments (*n* = 8), and of all the microorganisms tested *P. aeruginosa* was the most susceptible to the AMP activity of cyclized amylin (MBC = 0.4 µg/mL). See also Figure S1. **(E–H, J)** Spot tests were carried out by adding 50 µL of undiluted tube suspensions after 1 h incubation at 37^°^C. Spots are demarcated within separate circles labeled 1–8 representing the following amylin concentrations: 50 µg/mL, 25 µg/mL, 12.5 µg/mL, 6.25 µg/mL, 3.125 µg/mL, 1.6 µg/mL, 0.8 µg/mL, and 0.4 µg/mL. CONTROL untreated suspensions are spotted roughly around the center of the plate. (**K, L**) Time‐kill assays using a fixed amylin concentration of 10 µg/mL, and viable numbers of bacterial/yeast cells at 5, 10, and 15 min following incubation at 37^°^C at 80 rpm are represented here. A 3‐log reduction (≥99.9% killing) for *P. aeruginosa*, and a 4‐log reduction (≥99.99% killing) for *L. monocytogenes* are observed at 5 min. All microorganisms tested show at least a 3‐log reduction (≥99.9% killing) at 30 min post‐challenge. CFU (colony forming units); MBC (minimum bactericidal concentration).

We first tested synthetic cyclized amylin monomers for antimicrobial activity against the following clinically relevant bacteria and yeast: *Enterococcus faecalis*, *Pseudomonas aeruginosa*, *Streptococcus agalactiae*, *Listeria monocytogenes*, *S*. Typhimurium, *S. aureus*, and *C. albicans*. Dose‐dependent two‐fold amylin concentrations ranging from 100 µg/mL to 0.4 µg/mL were challenged with ≈2.5–5 × 10^5^ CFU/mL of the respective microorganism. A 3‐log reduction or ≥99.9% bacterial elimination was observed at 3.125 µg/mL for *E. faecalis*, *S. agalactiae*, and *L. monocytogenes* (Figure 1A, D, I). Of interest, *P. aeruginosa* was the most susceptible bacterial species, showing 99.9% elimination at 0.4 µg/mL (Figure 1B). A 3‐log reduction for *C. albicans* was observed at 12.5 µg/mL (Figure 1C). Spot tests from peptide‐microbe suspensions that were used to determine CFUs correlated with observed log reduction values (Figure 1E–H, J).

We next assessed the killing kinetics of cyclized amylin in a time‐kill assay at 5, 15, and 30 min, using 10 µg/mL peptide concentration. A 3‐log reduction (≥99.9% killing) for *P. aeruginosa* and a 4‐log reduction (≥99.99% killing) for *L. monocytogenes* were observed at 5 min (Figure 1K–L). Although at least 99% of *C. albicans* and *S. agalactiae* were eliminated in 5 min, 99% of *E. faecalis* elimination occurred in 15 min (Figure 1K, L). At the end of the 30‐min incubation, each of the test microorganisms showed at least a 3‐log reduction (≥99.9% killing), with *L. monocytogenes* exhibiting the most susceptibility with a 4.7 log reduction (≥99.99% killing) at 30 min (Figure 1K, L).

To test whether amino acid changes could alter the antimicrobial efficacy of amylin, reverse amylin peptide was tested against the same microorganisms in two‐fold dose‐dependent concentrations identical to those used for cyclic monomers. Although reverse amylin retained some antimicrobial effect at very high concentrations, its antimicrobial efficacy declined with decreasing concentrations compared to cyclic monomers (Figure S1A–E), suggesting that both amino acid sequence and cyclization influence the AMP activity of amylin. Bright‐field microscope images of bacteria and yeast post‐amylin or vehicle treatment resulted in clumps of aggregates within minutes in post‐amylin treatment (Figure S2A–E) but not in vehicle treatment samples (Figure S2F–J)

### 3.2 Scanning electron microscopy (SEM) and transmission electron microscopy (TEM) images reveal bacteriostatic and fungistatic entrapment mechanisms by amylin‐mediated peptide fibrilization

**FIGURE 2:**
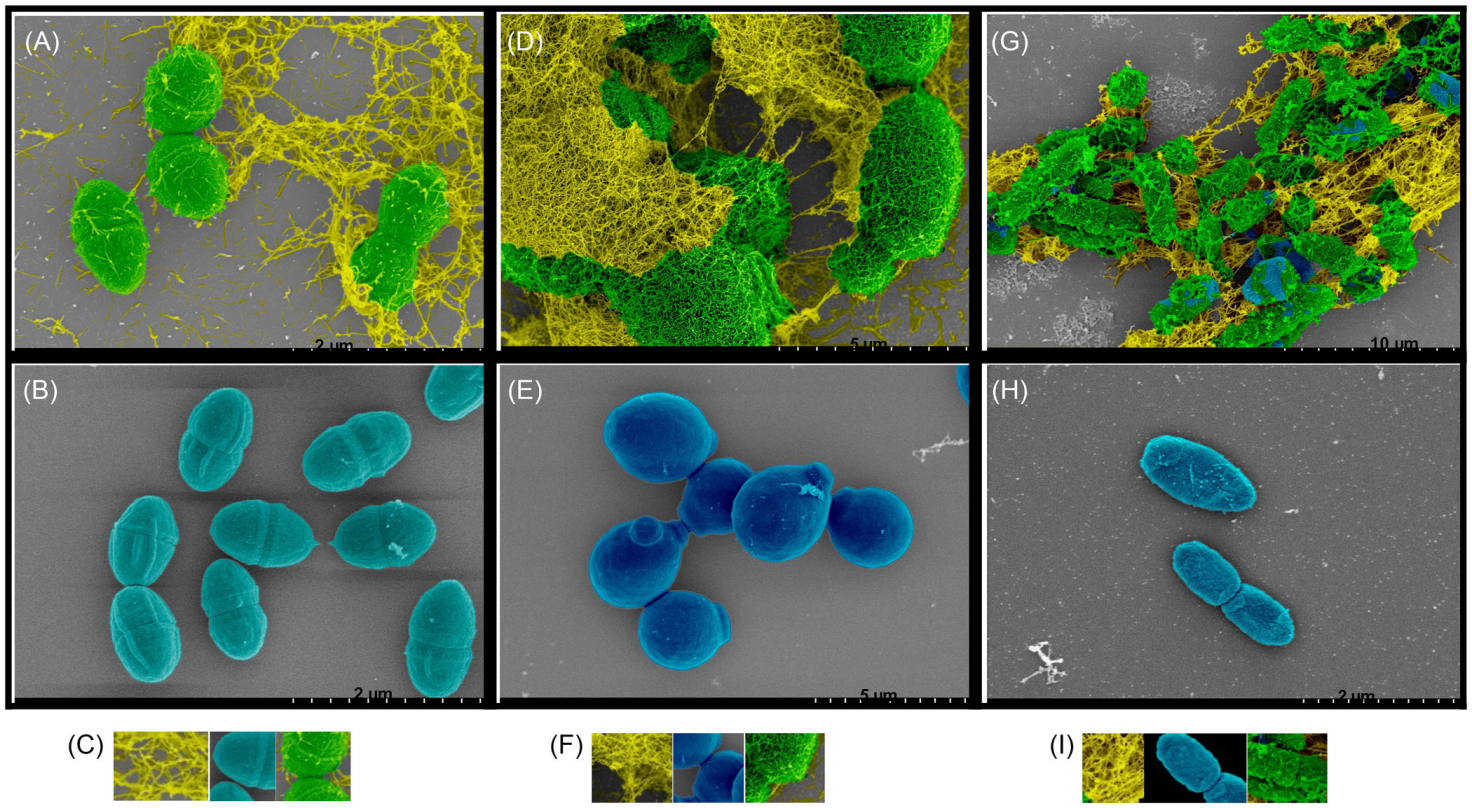
**Scanning electron microscopy of bacterial and yeast surfaces reveal amylin‐mediated microbial entrapment by peptide fibrilization (A, D, G, J, M)**: SEM captured after challenging 5 µg/mL synthetic cyclized monomeric amylin with three different microbes reveal fibril‐mediated entrapment stemming from amylin peptide binding to bacteria and yeast cell surface. **(A, D, G)** GREEN represents co‐localized regions between amylin fibrils (YELLOW) and bacterial or yeast cells (*Enterococcus faecalis*, *Candida albicans*, and *Pseudomonas aeruginosa*) represented by different shades of BLUE. **(B, E, H)** Control (PBS‐treated) bacteria and yeast show smooth cell surface texture and remain largely unaggregated. **(C, F, I)** Pseudo‐color representations are as follows: YELLOW represents amylin fibrils **(left panels of C, F, I)**, different shades of BLUE represent *E. faecalis*, *C. albicans*, and *P. aeruginosa* **(center panels of C, F, I)**. GREEN represents *E. faecalis*, *C. albicans*, and *P. aeruginosa* entrapped or co‐localized within amylin fibrils **(right panels C, F, I)**. See also Figures S2 & S3. SEM (scanning electron micrograph).

**FIGURE 3:**
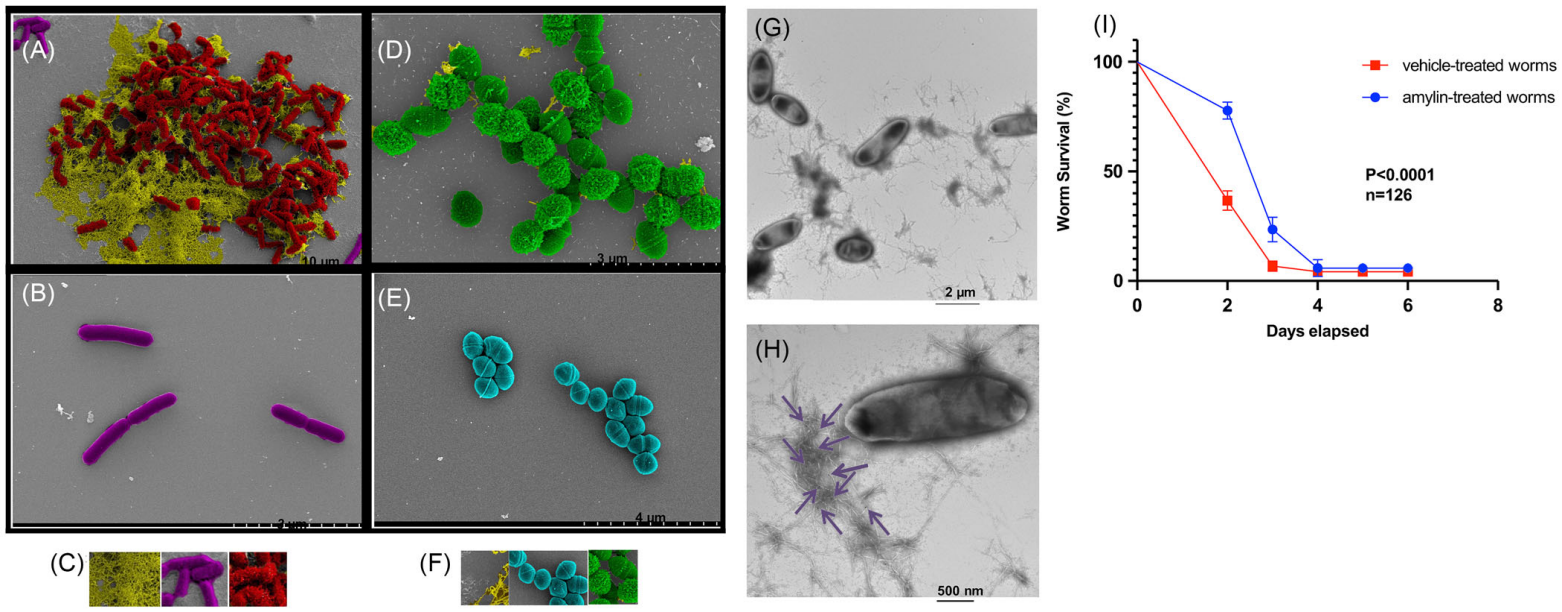
**SEM and TEM reveal that bacterial surface‐bound amylin‐mediated fibrils and amylin‐mediated antimicrobial actions confer host protection of *Caenorhabditis elegans* against *Candida albicans*. (A, D)** SEM captured after challenging 5 µg/mL synthetic cyclized monomeric amylin with two different bacteria reveal fibril‐mediated entrapment stemming from amylin peptide binding to bacterial surface. **(A)** RED represents co‐localized regions between amylin fibrils (YELLOW) and the cell surface of the bacterium *Listeria monocytogenes* (PURPLE). **(D)** GREEN represents co‐localized regions between amylin fibrils (YELLOW) and the bacterium *Streptococcus agalactiae* (BLUE). **(B, E)** Control (PBS‐treated) bacteria show smooth cell surface texture and remain largely unaggregated. **(C, F)** Pseudo‐color representations are as follows: YELLOW represents amylin fibrils **(left panels of C and E)**, PURPLE represents *L. monocytogenes* **(center panel of C)**, RED represents *L. monocytogenes* entrapped or co‐localized within amylin fibrils **(right panel of C),** BLUE represents *S. agalactiae* **(center panel of F),** and GREEN represents *S. agalactiae* entrapped or colocalized within amylin fibrils **(right panels of F)**. **(G–H)** Representative lower and higher magnification images of *Salmonella* Typhimurium clumps processed for TEM and probed with anti‐amylin immunogold show *S*. Typhimurium entrapped in a web of fibrillar network immunoreactive to anti‐amylin immunogold (indicated by arrows). **(I)** A representative graph showing a survival curve demonstrating the amylin‐mediated rescue of *Candida albicans* infected by *Caenorhabditis elegans*. See also Figure S4B and C. Data are represented as mean ± standard error of the mean. Statistical analyses were done using the Mantel‐Cox and the Gehan‐Breslow‐Wilcoxon tests (^****^ *p* < 0.0001). SEM (scanning electron micrograph); TEM (transmission electron micrograph

Next, we used scanning electron microscopy (SEM) to reveal fibril‐mediated entrapment of bacteria and yeast by amylin deposits. Pseudo‐colored SEM reveals, in detail, compacted bacteria and yeast cells swathed in amylin peptide fibrils, with some bacterial cells showing signs of shrinkage, membrane damage, and imminent cell death compared to control PBS‐treated bacteria (Figure 2A–I and Figure 3A–F). Transmission electron micrographs (TEM) and SEM of *S*. Typhimurium and *S. aureus* aggregates revealed bacteria ensnared in amylin fibrils immunoreactive to anti‐amylin immunogold antibody (Figure 3G, H and Figure S3A–E) confirming amylin as the potentiating peptide source of the fibrils. Although *S*. Typhimurium and *S. aureus* were resistant to amylin's bactericidal action, they exhibited increased susceptibility to amylin's bacteriostatic cell entrapment mechanism (Figure 3G, H and Figure S3A–E). In summary, SEM and TEM visually underscore fibrilization as a critical facet underlying amylin‐mediated microbial entrapment and agglutination, further validating amylin as a human AMP.

### 3.3 Amylin‐mediated antimicrobial actions confer host protection of human brain neuroglioma (H4) cells and *C. elegans* against *S*. Typhimurium and *C. albicans*

Next, to further assess the bacteriostatic properties of amylin in vitro, we used an *S*. Typhimurium gentamycin invasion assay. This assay was performed to determine whether amylin confers host cell protection similar to human alpha‐defensin 6 (HD6)^35^ and Aβ, which can bind and entrap bacteria in a non‐microbiocidal manner. Human cell monolayers (HCMs) prepared from adherent immortalized human brain neuroglioma (H4) cell lines were used to test the efficacy of amylin to neutralize *Salmonella* invasion of the cell monolayers in a well‐established gentamycin protection assay.^32^, ^33^ H4 cells were infected with 2.5–5 × 10^7^ CFU *S*. Typhimurium, which has been pre‐treated with 10 µg/mL monomeric cyclized amylin or vehicle (water), followed by an enumeration of the total numbers of intracellular bacteria by gentamycin protection assay.^33^ The numbers of *S*. Typhimurium cells that adhered to and eventually invaded the H4 cells were significantly lower in cells infected with amylin‐pretreated *S*. Typhimurium, demonstrating almost 99% inhibition of pathogen invasion (Figure S4A, B). This assay highlights the bacteriostatic properties of amylin to subjugate the host cell invasion despite the absence of bactericidal activity against *S*. Typhimurium.

*C. elegans* possess several mammalian orthologs that are evolutionarily conserved and constitute essential elements of the worm's innate immune system, making them a suitable model for testing antimicrobial functions of various compounds.^36^, ^37^, ^38^ In addition, it has been established previously that *C. albicans* ingestion leads to infection and death in *C. elegans*. Therefore, we used a modified version of an established *C. elegans* infection protocol^34^ to further explore the AMP activity of amylin. Worms were first fed with *C. albicans*, resulting in persistent infection of the worms with the yeast; the worms were then treated with 50 µg/mL amylin in 35 mm polypropylene dishes. After the treatment, the worms were monitored regularly for 6 days. Dead and dying nematodes were readily identified from the penetrative filamentation of the worm cuticle by *C. albicans* hyphae caused by extensive *C. albicans* proliferation in the worm gut (Figure S4C). Infected nematodes were significantly rescued following amylin treatment compared to the untreated worms (Figure 3I and Figure S4D).

### 3.4 Combinatorial concentrations of synthetic amylin and Aβ42 show synergistic antimicrobial activity against *S*. Typhimurium and *S. aureus*

Despite previous studies regarding the role of amylin in AD pathogenesis, the amylin‐Aβ interaction has not been explored in detail.^24^ Cross‐seeding of amylin and Aβ as a possible catalyst for neurodegenerative pathology observed in AD has been proposed previously.^28^, ^29^, ^30^ In addition, amylin has been reported to co‐localize with Aβ in amyloid plaques in the post‐mortem brains of AD patients, including those without the co‐morbidity of T2D.^24^ These findings suggest the possibility of concerted coordination between amylin and Aβ in potentiating pathogenic pathways in AD or host defense. Thus, we next tested whether amylin and Aβ may cooperate as antimicrobial peptides.

**FIGURE 4:**
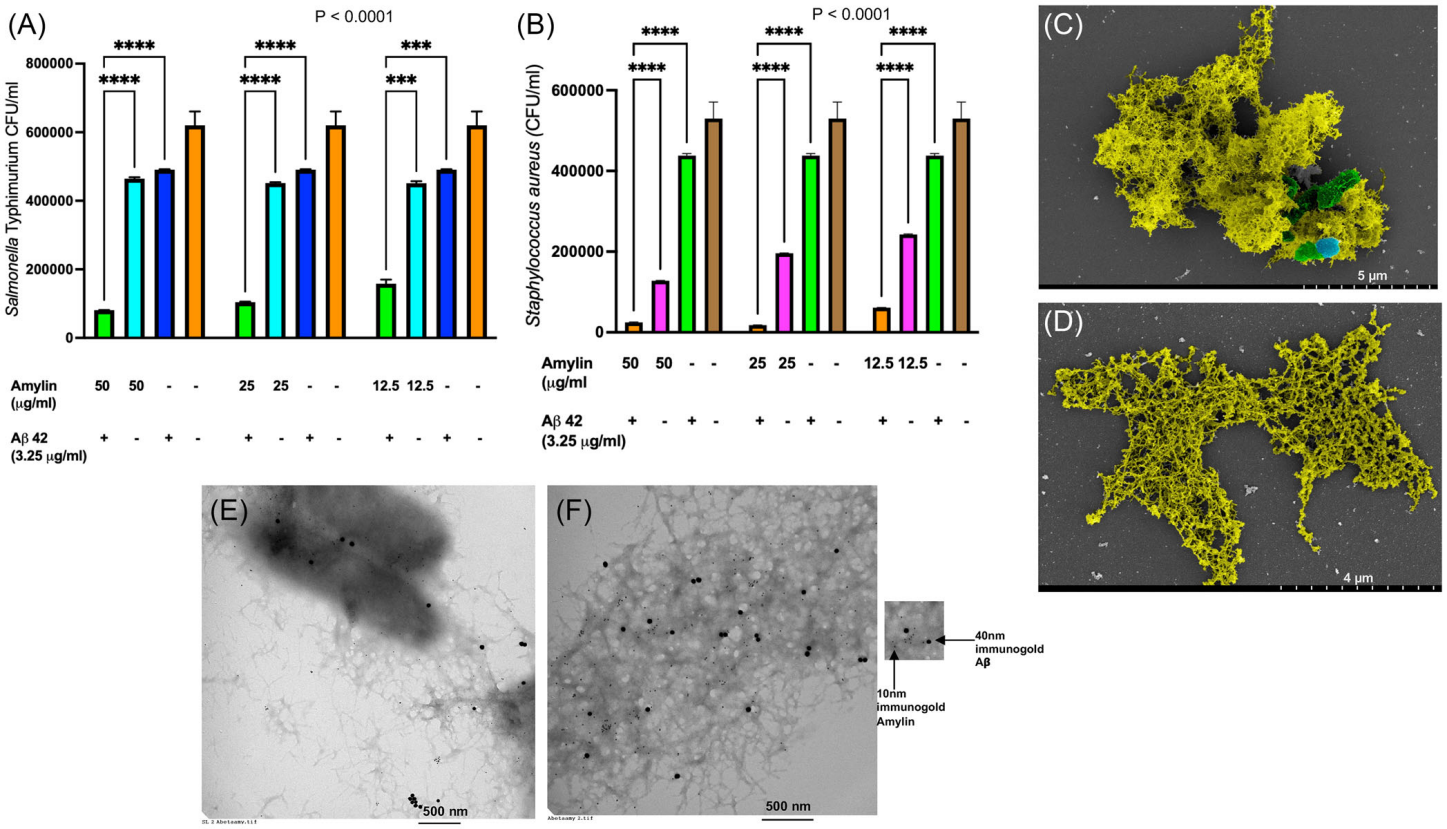
**Combinatorial concentrations of synthetic amylin and Aβ42 show synergistic antimicrobial activity against *Salmonella* Typhimurium and *Staphylococcus aureus*, and co‐localized/intertwined amylin and Aβ42 fibrils entrapment of both bacteria as observed in SEM and TEM images. (A, B)** Bacterial enumeration of viable count in CFU/mL after amylin and Aβ in combination or standalone doses—amylin: Aβ 42 (50 µg/mL:3.25 µg/mL, 25 µg/mL:3.25 µg/mL and 12.5 µg/mL:3.25 µg/mL), amylin (50 µg/mL, 25 µg/mL and 12.5 µg/mL), and Aβ42 (3.25 µg/mL)—were challenged with ≈2.5–5 × 10^5^ CFU/mL of *S*. Typhimurium or *S. aureus*. CFU numbers were obtained from five biological replicates for each condition, and statistical analyses were done using 2‐way ANOVA (^****^ *p* < 0.0001). Data are represented as mean ± standard error of the mean. **(C,D)** Pseudo‐colored SEM (YELLOW = amylin/Aβ42 fibrils, BLUE = *S*. Typhimurium, and GREEN = *S*. Typhimurium entrapped in amylin/Aβ42 fibrils). **(E, F)** TEM shows *S*. Typhimurium bacilli entangled and tightly enmeshed in fibrils immunoreactive to 10 nm (specific for anti‐amylin primary antibody) and 40 nm (specific for anti‐Aβ primary antibody) immunogold secondary antibodies. SEM (scanning electron micrograph); TEM (transmission electron micrograph); CFU (colony forming units)

For this purpose, we challenged ≈2.5–5 × 10^5^ CFU/mL of *S*. Typhimurium or *S. aureus* with different combinatorial and stand‐alone synthetic amylin and Aβ42 at various concentrations—amylin (50 µg/mL, 25 µg/mL, and 12.5 µg/mL), Aβ42 (3.25 µg/mL), and amylin:Aβ42 (50 µg/mL:3.25 µg/mL, 25 µg/mL:3.25 µg/mL, and 12.5 µg/mL:3.25 µg/mL). We chose these bacterial species because both have been demonstrated to be resistant to amylin's antimicrobial (bactericidal) effects. Bacterial enumeration of viable counts in CFU/mL revealed that amylin and Aβ42 in combination collectively enhanced bactericidal activity against *S*. Typhimurium and *S. aureus* (Figure 4A, B) suggesting a plausible antimicrobial synergistic relationship between these two amyloidogenic peptides. The CFUs of *S*. Typhimurium and *S. aureus* showed a significant reduction upon treatment with amylin and Aβ42 combo compared to treatments using either amylin or Aβ42 alone.

The combination of amylin and Aβ42 led to a dual bacteriostatic and bactericidal mode of action against *S*. Typhimurium and *S. aureus* (Figure 4A‐B). Pseudo‐colored SEM and TEM images revealed *S*. Typhimurium bacilli entangled and tightly enmeshed in fibrils as before. The intertwined amyloid fibrils were immunoreactive to both amylin and Aβ immunogold antibodies. Therefore, the fibrils originated from both amylin and Aβ42 as revealed by TEM with immunogold staining using 10 nm (specific for anti‐amylin primary antibody) and 40 nm (specific for anti‐ Aβ primary antibody) immunogold secondary antibodies, which co‐localized within amyloid fibril aggregates with entrapped *S*. Typhimurium (Figure 4C–F).

### 3.5 Human 3D neural cell cultures and primary astrocytes secrete amylin following infection with *Salmonella* Typhimurium

**FIGURE 5:**
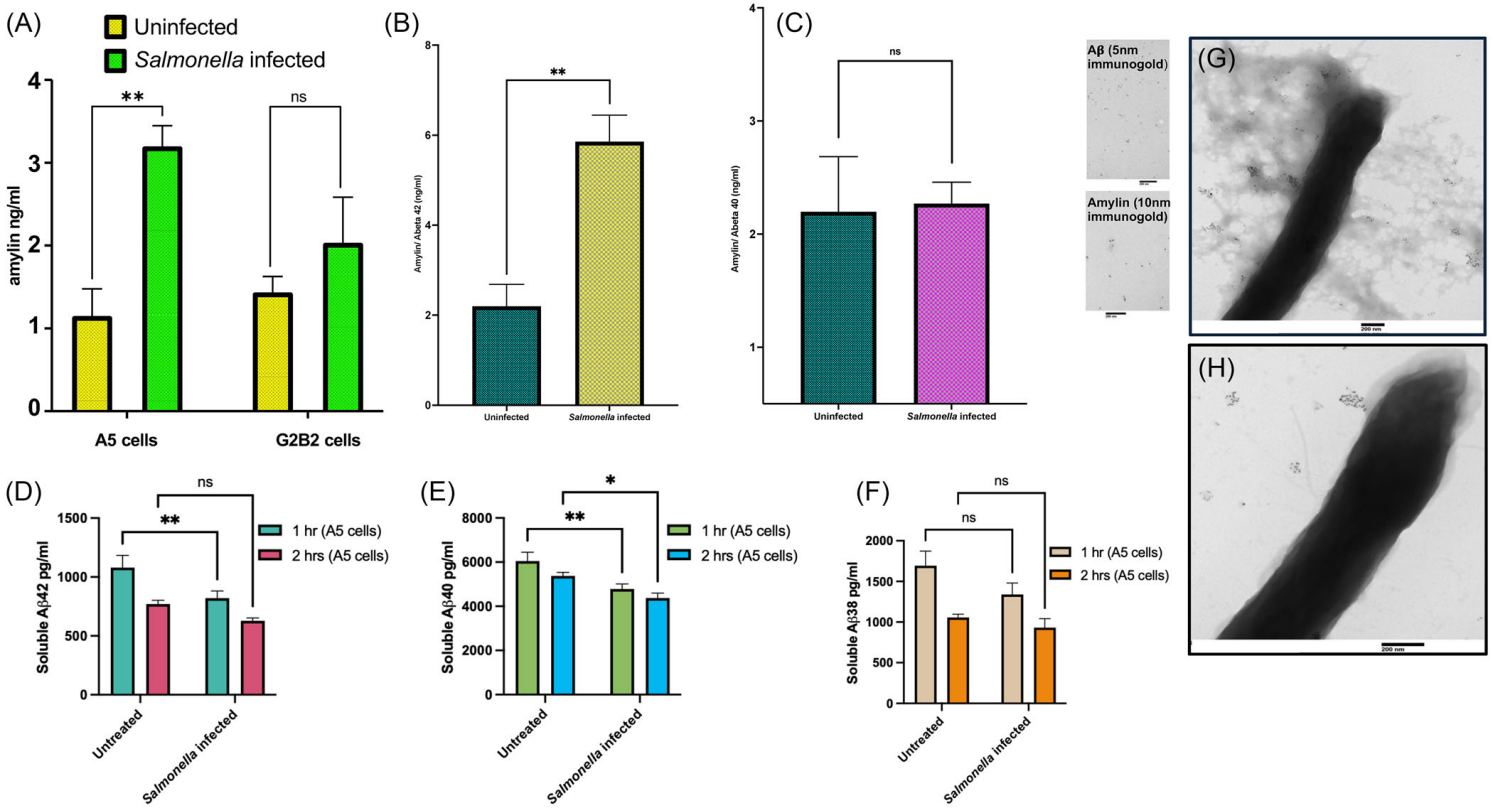
***Salmonella* Typhimurium infection in human three‐dimensional (3D) neural cell cultures alters amylin secretion and Aβ isoform concentrations (Aβ42, Aβ40, Aβ38), accompanied by changes in amylin/Aβ42 and amylin/Aβ40 ratios. (A)** A **r**epresentative graph with data from an ELISA assay specific to secreted human amylin in supernatants was collected from A5 (Aβ‐producing) and G2B2 (control) cells 18–24 h post‐infection. Representative graph with data comparisons between uninfected and infected A5 and G2B2 cells. Data are represented as mean ± standard error of the mean. Statistical analyses were carried out by a two‐tailed unpaired *t*‐test (^**^ *p* = 0.0025). **(B)** A representative graph with ELISA‐based analysis of amylin: Aβ42 ratio from supernatants collected from A5 (Aβ‐producing) cells 18–24 h post‐infection. The graph compares uninfected and infected A5 cells. Data are represented as mean ± standard error of the mean. Statistical analyses were carried out by a two‐tailed unpaired *t*‐test with Welch's correction (^**^ *p* = 0.0033). **(C)** A representative graph with ELISA‐based analysis of amylin: Aβ40 ratio from supernatants collected from A5 (Aβ‐producing) cells 18–24 h post‐infection. The graph compares uninfected and infected A5 cells. Data are represented as mean ± standard error of the mean. Statistical analyses were carried out by a two‐tailed unpaired *t*‐test with Welch's correction (*p* = 0.8975). **(D)** A representative graph shows soluble Aβ42 from supernatants of uninfected and infected A5 (Aβ‐producing) after 1 h and 2 h post‐*S*. Typhimurium infection. Data are represented as mean ± standard error of the mean. Statistical analyses were carried out by two‐way ANOVA (^*^ *p* = 0.0052). **(E)** A representative graph shows soluble Aβ40 from supernatants of uninfected and infected A5 (Aβ‐producing) after 1 h and 2 h post‐*S*. Typhimurium infection. Data are represented as mean ± standard error of the mean. Statistical analyses were carried out by two‐way ANOVA (^**^ *p* = 0.0223 & ^*^ *p* = 0.0039). **(F)** A representative graph shows soluble Aβ38 from supernatants of uninfected and infected A5 (Aβ‐producing) after 1 h and 2 h post‐*S*. Typhimurium infection. Data are represented as mean ± standard error of the mean. Statistical analyses were done by two‐way ANOVA (1 h *p* = 0.1350). **(G, H)** TEM shows *S*. Typhimurium bacilli entangled and tightly enmeshed in amyloid aggregates and fibrils immunoreactive to 10 nm (specific for anti‐amylin primary antibody) and 5 nm (specific for anti‐Aβ primary antibody) immunogold secondary antibodies.

Amylin is present in the brain; however, data supporting the synthesis of this peptide remain unclear.^29^ Some studies have asserted that amylin is not synthesized in the brain but transported there by blood following secretion from pancreatic cells.^24^ However, amylin messenger RNA (mRNA) has also been reported previously in the brain and astrocytes.^39^, ^40^ We addressed this discrepancy using our previously reported 3D human neural cell culture model of AD that overexpresses Aβ (A5) and the control cell line (G2B2).^41^ The 3D cells, comprising ≈70% neurons, ≈30% astrocytes, and ≈1% oligodendrocytes, were infected with *S*. Typhimurium overnight (18–`24 h) containing a low concentration of gentamycin (10 µg/mL) in the cell media to control bacterial‐induced host cell death. ELISA of supernatants collected after overnight incubation (18–24 h after infection) revealed increased levels of human amylin in both the A5 and G2B2 cells following infection with *S*. Typhimurium. The increase in secreted amylin levels was significantly higher in infected A5 cell supernatants versus uninfected A5 cells (Figure 5A).

Given the complexity and cell type diversity that make up the 3D cell line, we next used primary human astrocytes to narrow down the candidate cell type from which amylin was possibly secreted following infection. Human primary astrocytes were infected with *S*. Typhimurium for 1 h and 2 h, and the supernatants were screened for human amylin by ELISA. We observed a significant increase in amylin levels at 2 h post‐*S*. Typhimurium infection but not at 1 h (Figure S5A). Data obtained from reverse transcription ‐polymerase chain reaction (PCR) showed that *S*. Typhimurium infection of astrocytes led to significantly increased levels of amylin mRNA at 1 h and 2 h post‐infection as compared to vehicle‐treated control, thereby confirming observations obtained from 3D cell line infection experiments (Figure S5B). Amylin protein levels continued to increase 18–24 h post‐infection of astrocytes as compared to 1 h post‐infection (Figure S6A).

To assess how *S*. Typhimurium infection in 3D cells influences the balance between amylin and Aβ isoforms with known antimicrobial properties, we quantified the relative levels of amylin, Aβ42, and Aβ40 following infection. Ratios of amylin to Aβ42 and Aβ40 were evaluated to determine the potential shifts in Aβ peptide isoform dynamics. Post‐infection, the amylin/Aβ42 ratio showed a significant increase compared to the amylin/Aβ40 ratio, which remained statistically unchanged (Figure 5B‐C). These changes in the amylin/Aβ42 and amylin/Aβ40 ratios suggest that infection leads to differential regulation of Aβ peptide species, possibly favoring a relative increase in Aβ40 or decrease in Aβ42, and altering amylin dynamics. Because Aβ42 is more aggregation‐prone and neurotoxic and is more closely associated with AD, and given its stronger antimicrobial activity than Aβ40, this could suggest antimicrobial‐induced aggregation of Aβ42 at infection sites, lowering its measurable levels, or it could be due to deliberate shift in the peptide response, with amylin being more dominant in the antimicrobial role in this context. Aβ40 is less prone to aggregation than Aβ42; as a result, the host may upregulate Aβ40 to avoid toxicity while still mounting an effective antimicrobial response, possibly as a safer, less aggregation‐prone antimicrobial agent. This implies a coordinated shift in antimicrobial peptide isoform balance, potentially to optimize defense while minimizing tissue damage associated with amyloid aggregation.

Furthermore, cell culture supernatants collected 1 and 2 h after *S*. Typhimurium infection of A5 cells showed a marked reduction in soluble isoforms Aβ40 and Aβ42, indicating their aggregation through antimicrobial activity (Figure 5D, E). Although Aβ38 showed a downward trend, this decrease was not statistically significant (Figure 5F). Co‐immunogold staining of the pelleted *S*. Typhimurium collected 2 h after A5 cell infection revealed large amyloid aggregates that were positive for both amylin and Aβ in TEM images. This supports the hypothesis that the reduction in soluble Aβ40 and Aβ42 resulted from their combined antimicrobial action with cell‐derived amylin that binds to and traps bacteria, producing pelleted debris (Figure 5G).

TEM images of pelleted *S*. Typhimurium from G2B2 cells displayed smaller amyloid aggregation and less amylin/Aβ co‐localization (Figure 5H). It is important to note that the significant increase in astrocyte amylin levels (Figure 5C) aligns with the corresponding reduction in Aβ40 and Aβ42 observed at 1 h and 2 h post‐infection of A5 cells. This further supports the observation of a combined antimicrobial‐induced aggregation of amylin, Aβ40, and Aβ42 at 1 h and 2 h after *S*. Typhimurium infection. In addition, immunostaining with an anti‐amylin antibody revealed intracellular amylin 1 h post‐infection in astrocytes, compared to PBS‐treated astrocytes (Figure S6B, C), further validating the above findings. These results suggest that amylin levels in the 3D neural cell cultures primarily originate from astrocytes. The combined data obtained from challenging 3D neural cell cultures or human primary astrocytes with *S*. Typhimurium demonstrate, for the first time, the antimicrobial synergism between two of the most well‐established, pathogenic amyloid‐prone proteins: amylin and Aβ.

We performed histological co‐staining using antibodies against amylin and bacterial markers to assess the potential microbial interactions on post‐mortem human AD brains. Distinct amylin antibodies were used for Gram‐positive and Gram‐negative bacterial targets to ensure staining specificity. We observed prominent co‐localization of amylin with Gram‐positive bacteria (Figure S7A–C). Chromogenic detection was performed using DAB (brown) or Ventana Purple, and sections were counterstained with hematoxylin. Brightfield microscopy revealed a strong amylin signal colocalizing with Gram‐positive bacteria in multiple areas, showing strong, moderate, and weak signal overlap between amylin and Gram‐positive markers, supporting microbial association in AD pathology.

Histological findings support our central hypothesis that amylin accumulates in response to bacterial infection in the AD brain. They also parallel our experimental infection models using *S. aureus* and *S*. Typhimurium as representative Gram‐positive and Gram‐negative pathogens, respectively.

### 3.6 Proposed model for antimicrobial activities of soluble amylin in synthetic and cell‐derived forms both as a standalone antimicrobial peptide (AMP) and in conjunction with synthetic or cell‐derived Aβ isoforms

**FIGURE 6:**
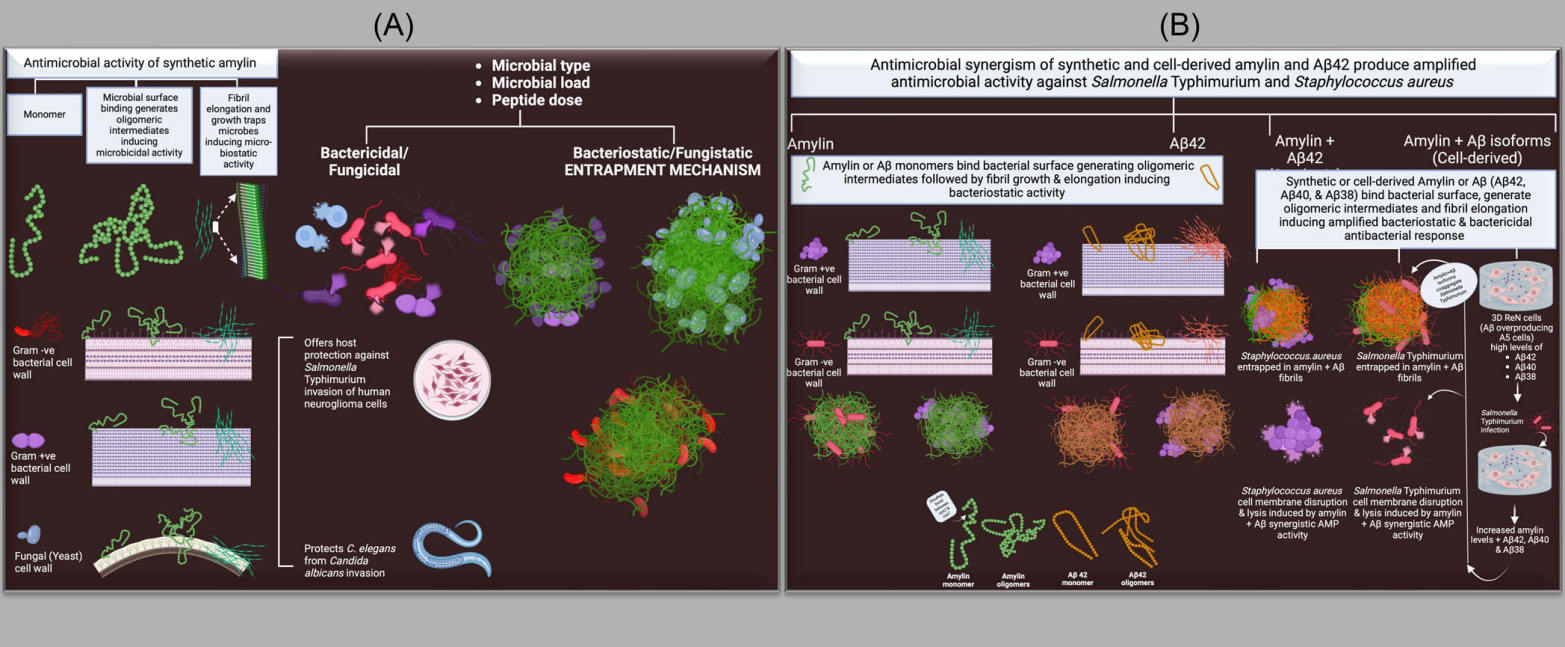
**Proposed model for antimicrobial activities of soluble amylin in synthetic and cell‐derived forms both as a standalone antimicrobial peptide (AMP) and in conjunction with synthetic or cell‐derived Aβ isoforms. (A, B)**. The suggested mechanism involves microbial cell surface binding, causing agglutination and entrapment, which leads to microbiostatic or microbicidal effects of amylin alone or in combination with Aβ. The antimicrobial effect of amylin also confers host protection in human neuroglioma cells and *Caenorhabditis elegans* against *Salmonella* Typhimurium or *Candida albicans*. “Created with ‘BioRender.com.”

Colors representing Gram +ve, Gram –ve, and yeasts are purple, pink, and blue, respectively. Based on the details obtained from electron microscopy images, the entrapped microbes appeared in different states, ranging from viable but weakened to dead or dying. Therefore, to reflect these observations, brighter purple, pink, or blue shades represent viable but weakened cells. In comparison, darker shades represent dead/dying microbial cells (Figure 6A). In conclusion, the collective findings, as illustrated, propose a model for understanding the antimicrobial efficacy of amylin and the previously unreported amyloidogenic antimicrobial synergism between amylin and Aβ (Figure 6A, B).

## 4 DISCUSSION

Our findings highlight amylin's bactericidal, fungicidal, and microbiostatic activities against various clinically relevant microbes. Amylin's broad‐spectrum efficacy likely stems from its strongly cationic nature, which promotes high‐affinity binding to negatively charged bacterial surfaces.^42^, ^43^ Time‐kill assays show amylin's elimination of all test strains within 5 to 15 min, confirming amylin's swift antimicrobial activity. SEM and TEM confirm that amylin fibrils can entrap bacteria and yeast in a tough, protease‐resistant fibrillar meshwork. This is the first report of the complex antimicrobial association between amylin+/Aβ+ fibrils with dividing bacteria and budding yeast. The H4 cell invasion and in vivo *C. elegans* rescue studies underscore amylin's multifaceted AMP activity, likely mediated by physicochemical properties common to other human AMPs.^17^, ^44^, ^45^, ^46^, ^47^ Finally, combinatorial infection of 3D neural cell cultures or human primary astrocytes with *S*. Typhimurium provides the first evidence of antimicrobial synergism between amylin and Aβ.

AMPs act through both bactericidal mechanisms, such as membrane disruption and ribosomal inhibition, as well as bacteriostatic mechanisms, including immobilizing pathogens within fibrillar networks. Our findings demonstrate that amylin exhibits both strategies: It rapidly kills a variety of microbes through direct contact (spot tests and time‐kill assays) while also forming fibrils that entrap bacteria and fungi. This dual action not only underscores the potency of amylin as an AMP but also links it as an innate immune trigger toward amyloid plaque formation in AD and T2D.

Combining multiple mechanisms of action, AMPs can mediate a broad and complex range of innate immune responses depending on a pathogen's characteristics and behavior. This allows AMPs to behave dynamically, inducing tailored AMP‐mediated immunomodulatory activities necessary for enhanced host protection that is as crucial as the peptides’ direct antimicrobial actions.^48^, ^49^, ^50^ This contrasts starkly with traditional antibiotics, which indiscriminately kill targeted and untargeted microbes, contributing to the irrepressible and unchecked development of drug resistance among common pathogens.^48^, ^49^, ^50^, ^51^, ^52^ From our observations, synthetic monomeric amylin appears to mimic or surpass the behavioral properties of other AMPs, such as LL‐37, Aβ, and HD 5/6. However, as with Aβ, despite the remarkable capacity of amylin to neutralize microbes, the physicochemical tendency of this peptide to aggressively aggregate may inexorably become deleterious to the cells/organs it was recruited to protect.

Peptides have been studied extensively as both bactericidal and bacteriostatic agents, playing a vital role in innate immunity. Classic antimicrobial peptides have been employed clinically since the 1940s.^53^ In addition, proteins like lactoferrin contribute to nascent immune defense through a dual mechanism involving iron sequestration and direct antimicrobial peptide activity.^54^ Our findings on amylin add to this rich literature, highlighting its multifunctional role in host defense.

Amyloidogenic AMPs like Aβ and amylin are part of a much larger innate defense system that includes the regulation of oxidative stress and the sequestration of heavy metals, particularly iron, which is essential for bacterial proliferation. Iron sequestration during infection deprives pathogens of a key nutrient. This is achieved partly by redirecting circulating iron into tissue stores via ferritin, a process upregulated during inflammation.^55^, ^56^, ^57^ APP itself is involved in this process through an iron‐responsive element (IRE) in its 5′ untranslated region^16^ and ferroxidase‐like activity that promotes iron export.^15^ Thus, APP and its cleavage product Aβ may contribute to innate immunity through both antimicrobial activity and regulation of iron homeostasis.

Aβ, like other vertebrate AMPs, displays a tremendous degree of pleiotropism, including microbicidal, microbiostatic, and immunomodulatory mechanisms. Furthermore, despite primary amino acid sequence differences, Aβ and amylin share some standard physicochemical features.^17^, ^58^ Based on our findings, amylin, like Aβ, binds to microbial surface moieties, resulting in microbicidal mechanisms depending on the targeted pathogen. In addition, like Aβ, amylin's propensity for forming fibrils and aggregating into amyloid plaques functions to entrap and immobilize pathogens, preventing their eventual spread and host invasion. Suffice it to say that there is a 50% sequence similarity and 25% identity between amylin and Aβ. Notably, the highest similarity is located within the β‐sheet regions associated with fibril formation,^27^, ^59^ a feature that incidentally is also key to the antimicrobial microbiostatic entrapment mechanism of Aβ and amylin.

Although amylin is primarily a pancreatic peptide, evidence of it in the central nervous system includes amylin mRNA and protein detection in astrocytes of the cerebral cortex and hippocampus, and transcriptomic profiling confirming *IAPP* expression in astrocytic populations.^24^, ^60^ Although expressed at lower levels compared to canonical astrocytic proteins such as APP, amylin expression may be upregulated under inflammatory or infectious conditions, potentially reaching physiologically relevant levels that allow for fibril formation and microbial entrapment.

Based on our investigation, we propose that amylin and Aβ are initially secreted as part of the host's innate immune response, potentially acting independently or synergistically to capture or neutralize invading pathogens at specific focal sites of infection, such as the brain and pancreas. However, the subsequent amyloid deposition then drives the pathogenic features of AD and T2D, respectively. Both Aβ and amylin oligomers bind to negatively charged lipids in cell membranes, disrupting the lipid bilayer and forming membrane pores, resulting in cell lysis. Aβ and amylin oligomers can further aggregate into fibrils, which are highly immunogenic in a toll‐like receptor (TLR)‐dependent manner, triggering the immune system and resulting in inappropriate inflammation.^61^, ^62^ The propensity of Aβ and amylin to form fibrils and aggregate into amyloid plaques is microbiostatic, a unique mechanism owing to their identity as amyloid proteins.^18^, ^19^, ^48^ It is an essential characteristic to consider when investigating amyloidogenic proteins for possible AMP status.

APP and amylin perform essential physiological functions. APP supports synaptic plasticity, neuronal growth, and metal ion homeostasis, while amylin regulates glucose, satiety, and calcium signaling in pancreatic and neural tissues.^63^ These roles are especially active during early postnatal development and persist into adulthood.

However, following infection, proteolytic processing may shift APP and amylin toward amyloidogenesis, resulting in fibrils that entrap microbes, mirroring other antimicrobial peptides in the innate immune system.^18^, ^19^ This dual‐function paradigm suggests that amyloidogenesis may have evolved partly as a protective host‐defense mechanism. Excessive or prolonged activation of this mechanism, however, may drive AD and T2D pathology.

In addition to directly observing antimicrobial activity in amylin, other studies have also demonstrated antimicrobial activity in amylin analogs and evolutionarily related peptides. Pramlintide, a synthetic amylin analog used to treat insulin resistance, has demonstrated fibrilization and antifungal activity in combination with zinc.^64^ Calcitonin gene‐related peptide (CGRP), an alternative splicing product of the calcitonin gene that belongs to the same family of peptides as amylin, has also shown antimicrobial activity.^65^, ^66^ Like amylin, CGRP, composed of 37 amino acids, is derived from the cleavage of a propeptide that is the product of the cleavage of a prepropeptide, which forms amyloids^67^ and is found in association with amylin plaques. In their human forms, CGRP and amylin show 43% amino acid sequence identity with amylin.

Aβ is a secondary proteinopathy in the pancreas in T2D and exhibits significant cross‐seeding with the dominant amylin plaques.^68^ Cross‐talk among amyloidogenic peptides in human diseases has been reported previously. For example, α‐synuclein of Parkinson's disease co‐aggregates with amylin in pancreatic β‐cell amyloid formation in T2D, and amylin‐tau cross‐seeding has been implicated in driving toxic tau pathology, raising the risk among individuals with T2D for AD.^69^, ^70^ Together with our findings, the above examples of cross‐talk between various amyloid‐prone peptides suggest an evolved host defense mechanism designed to enhance the host's antimicrobial response. Nonetheless, although AMP synergism may enhance immunity, it also risks promoting amyloid pathologies that trigger the disease. Thus, amylin‐Aβ antimicrobial synergism is a potential “double‐edged sword,” beneficial in fighting infection but possibly contributing to disease. Furthermore, pathogens such as *Porphyromonas gingivalis* and *C. pneumoniae* are observed in AD brains,^71^, ^72^, ^73^ supporting the notion that infections may be early triggers in disease progression.

To extend the translational significance of our findings, in the future, we will include multiplex staining on post‐mortem AD and T2D tissue. In this study, histological co‐staining using distinct amylin antibodies revealed robust co‐localization of amylin with Gram‐positive bacteria and weaker signals with Gram‐negative bacteria (data not shown), aligning with our in vitro models. These findings reinforce our hypothesis that amylin responds to microbial presence. It is notable that the detection of bacterial signals, regardless of species, is a significant pathological indicator in both AD and T2D, consistent with emerging infection‐driven amyloidogenesis. Future work on Aβ in triple staining protocols will expand to T2D brain tissue. Together, these next steps will help define the interplay between microbial signatures, amyloid peptides, and neuroinflammation across two major age‐related chronic diseases. Collectively, our findings suggest the hypothesis that microbes can trigger amylin‐Aβ cross‐seeding antimicrobial synergism and precipitate a cascade of innate immune responses, such as neurofibrillary tangles and neuroinflammation, that inexorably lead to the development of AD.

## CONFLICT OF INTEREST STATEMENET

The authors declare no conflicting interests. Any author disclosures are available in the Supporting Information

## CONSENT STATEMENT

All human subjects provided informed consent, or consent was unnecessary.

## Supporting information



Supporting Information

Supporting Information
