# Supplementary material for: Human amylin is a potent antimicrobial peptide that exhibits antimicrobial synergism with the amyloid beta protein
Source: Alzheimers Dement. 2025 Jul 29;21(8):e70490. doi: 10.1002/alz.70490 (PMC12307129; doi:10.1002/alz.70490)
Supplement: Supplementary file 1 — Supporting Information [file ALZ-21-e70490-s002.pdf]

Supplementary Figure 1

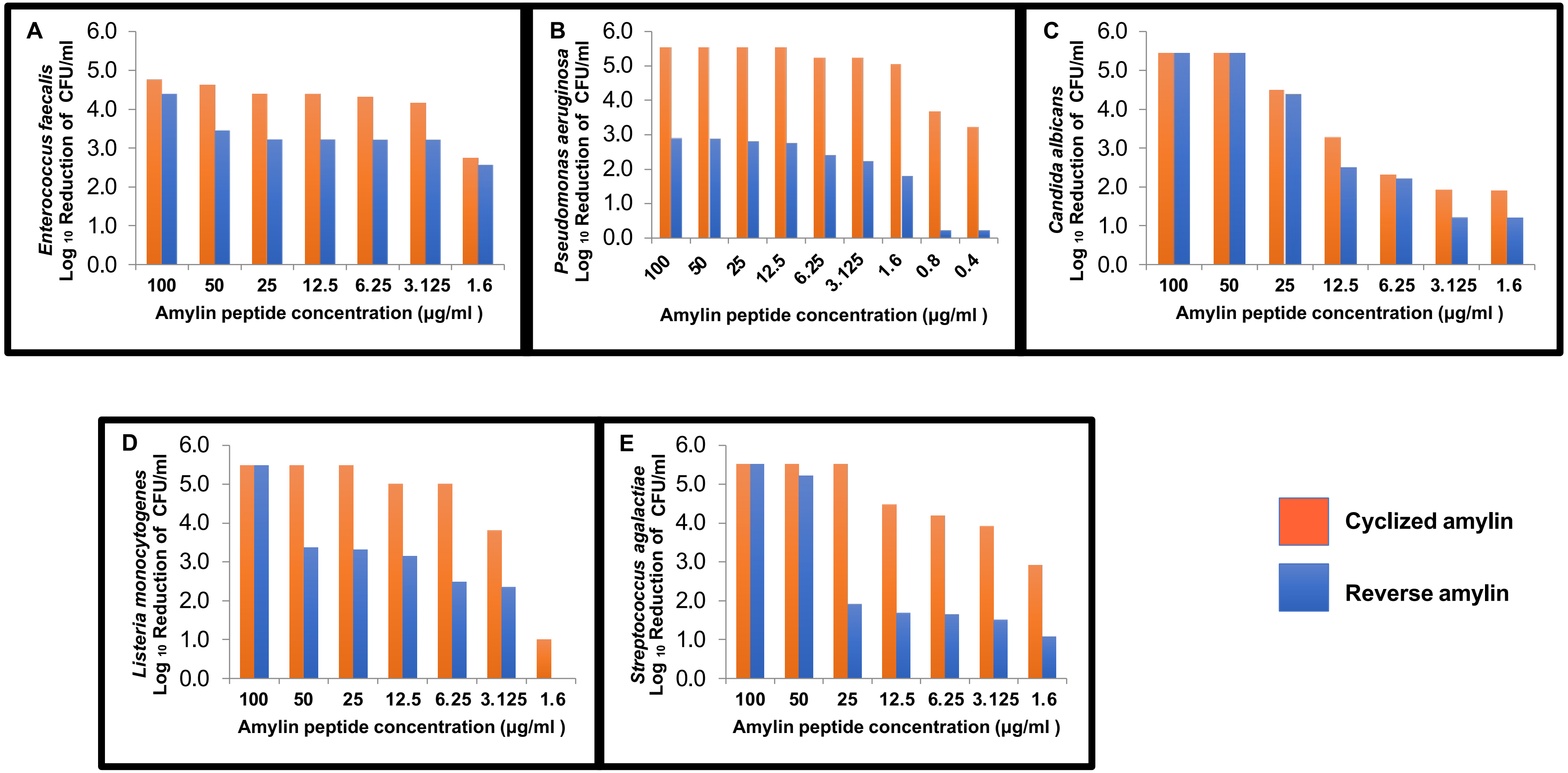

Supplementary Figure 1. Antimicrobial efficacy of cyclized versus reverse amylin in various microorganisms. A. *Enterococcus faecalis*, B. *Pseudomonas aeruginosa*, C. *Candida albicans, D. Listeria monocytogenes, and E. Streptococcus agalactiae*. Reverse amylin retains some antimicrobial effect at very high concentrations, but its antimicrobial efficacy markedly declines with decreasing concentrations compared to cyclic monomers. **(A-E)** Representative graphs comparing antimicrobial efficacies between cyclized and reverse amylin against the abovementioned microorganisms. The results are presented in $\log_{10}$ reduction of CFU/ml on the x-axis with a 3-log reduction $\geq$ 99.9% microbial elimination. CFU numbers were obtained from two independent experiments for every strain tested.

Supplementary Figure 2

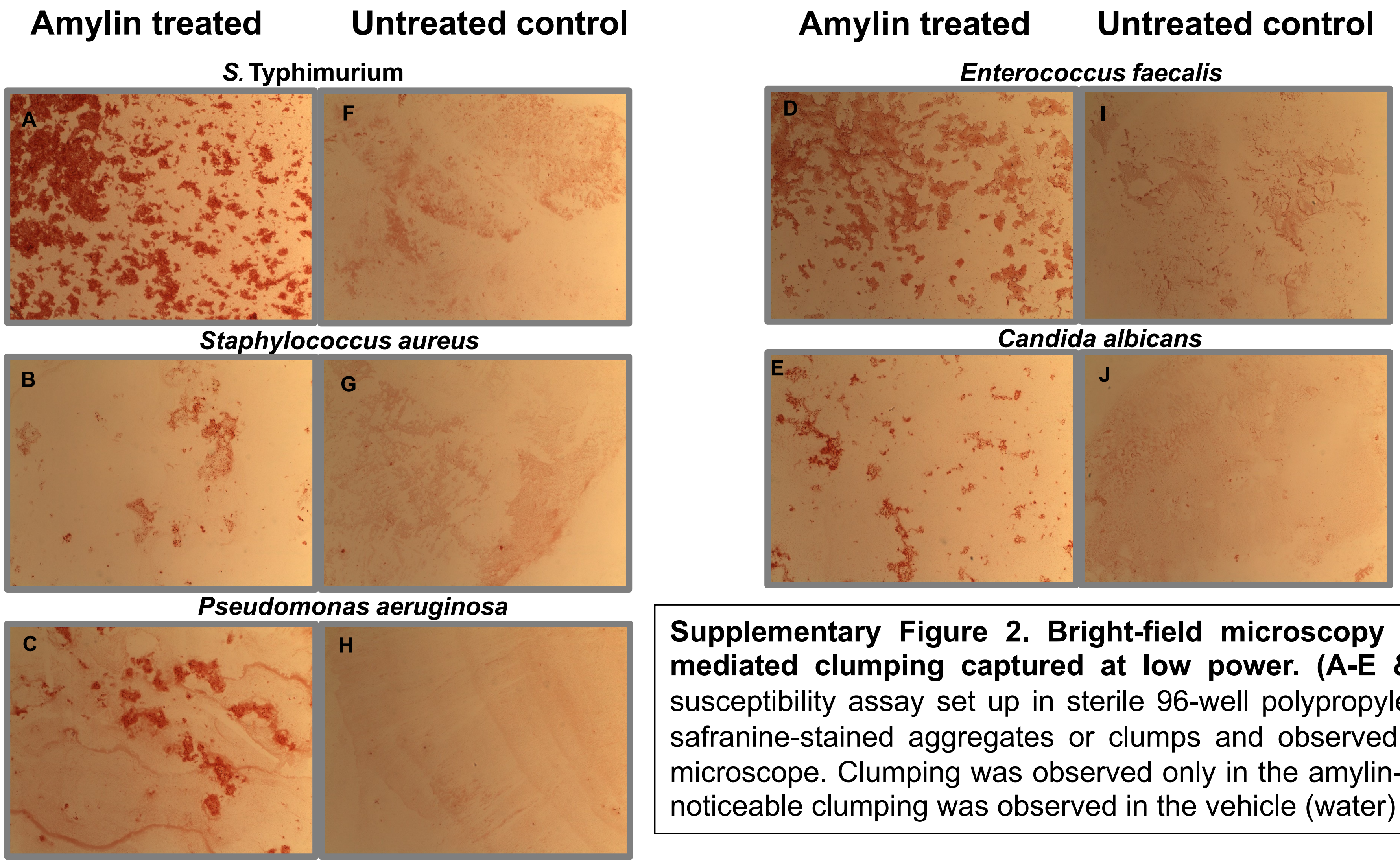

Supplementary Figure 2. Bright-field microscopy images of amylin-mediated clumping captured at low power. (A-E & F-J) Antimicrobial susceptibility assay set up in sterile 96-well polypropylene plates produced safranine-stained aggregates or clumps and observed under a bright-field microscope. Clumping was observed only in the amylin-treated samples. No noticeable clumping was observed in the vehicle (water) treated controls.

Supplementary Figure 3

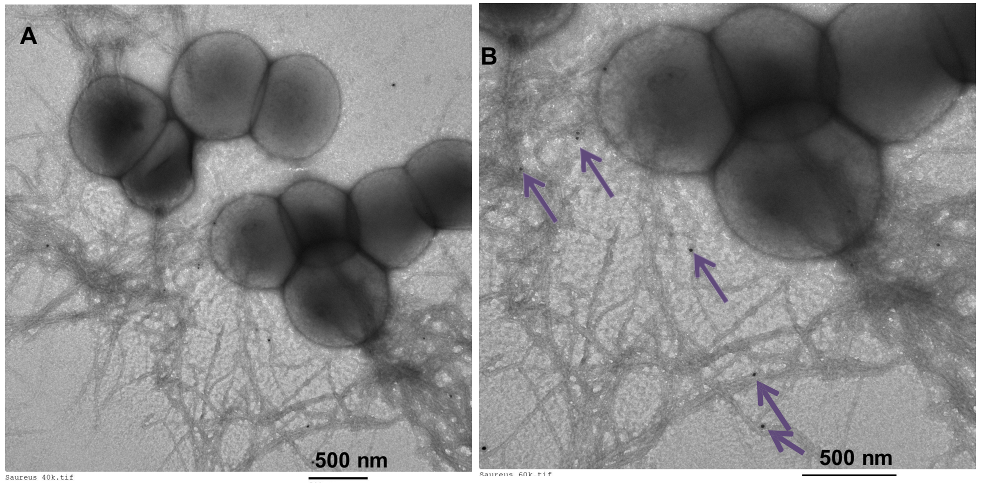

Supplementary Figure 3. TEM and SEM micrographs show that *Staphylococcus aureus* is susceptible to surface binding of cyclized amylin, leading to peptide-mediated fibrilization and microbial entrapment. **(A-B)** *S. aureus* clumps were processed for TEM, probed with anti-amylin immunogold, and imaged by TEM. Representative TEM figures show *S*. aureus entrapped in amylin fibrils at lower magnification. A higher magnification image confirms the interconnected fibrils to be immunoreactive to anti-amylin immunogold (indicated by arrows). **(C-E)** Scanning electron micrographs (SEM) were captured after challenging vehicle or 5μg/ml synthetic cyclized monomeric amylin with *S. aureus*. **(C)** Control (PBS-treated) *S. aureus* (PURPLE). **(D)** 5μg/ml synthetic cyclized monomeric amylin-treated *S. aureus* (RED represents colocalized regions between amylin fibrils (YELLOW) and *S. aureus* (PURPLE). **(E)** Pseudo colors: PURPLE (*S. aureus*), RED (*S. aureus* and amylin fibrils colocalized) , and YELLOW (amylin peptide fibrils).

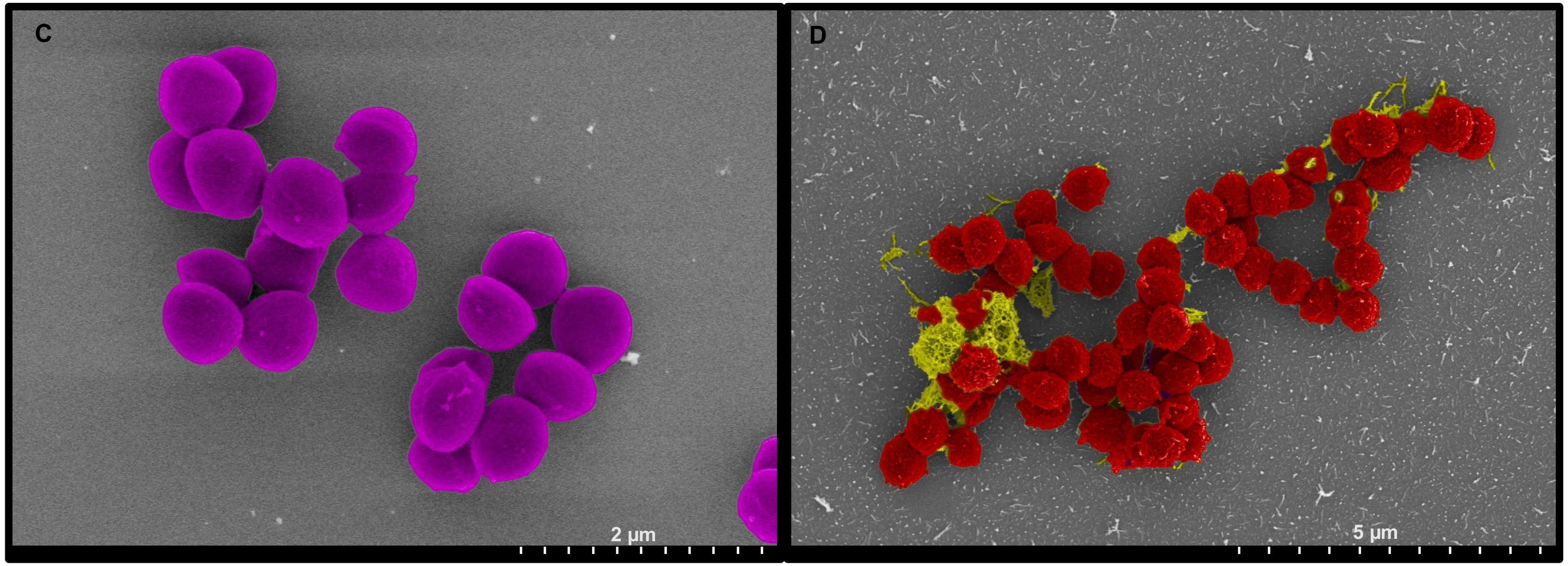

E

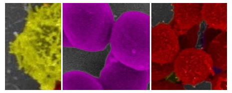

Supplementary Figure 4

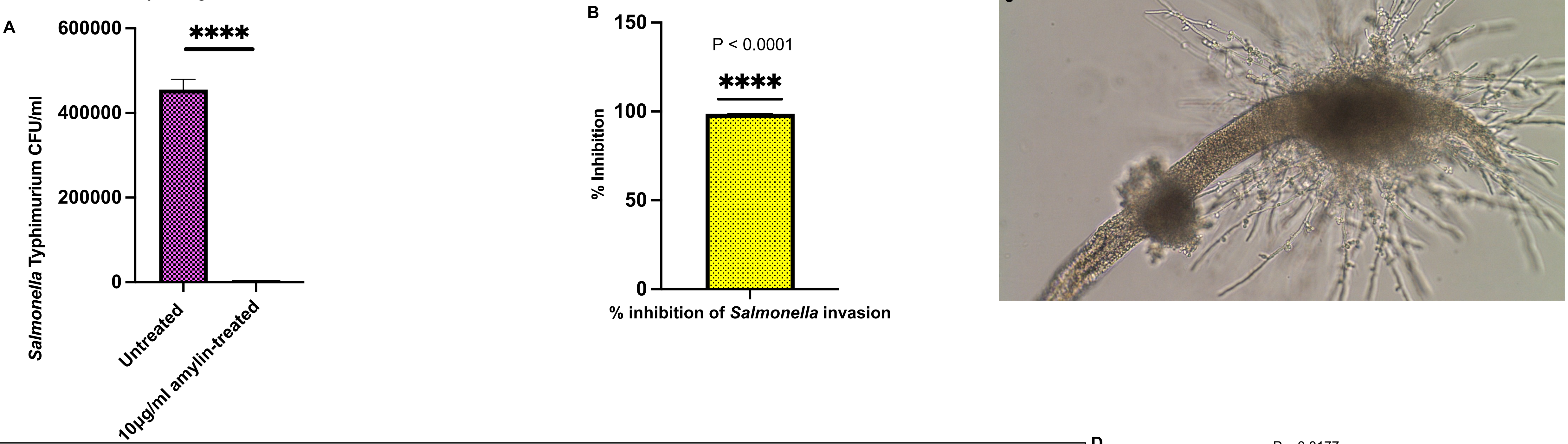

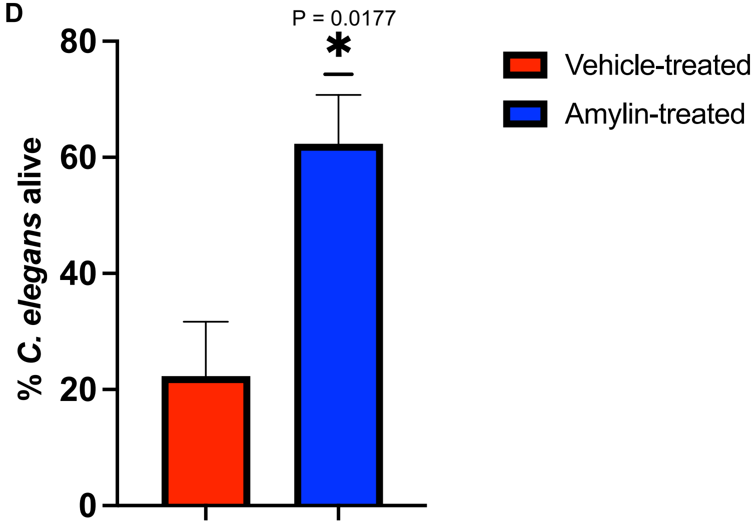

Supplementary Figure 4. Amylin treatment significantly rescued H4 cells from *Salmonella* Typhimurium invasion and *C. elegans* (N2 strain) infected with *Candida albicans*. Representative figures for amylin-mediated rescue of infected human neuroglioma (H4) cells from *Salmonella* Typhimurium invasion and *C. elegans* from *Candida albicans* infection. **(A)** Representative graph with results from the gentamycin protection assay following a 1 hr treatment of H4 cell monolayers with S. Typhimurium at 37°C in 5% CO2. The data show significantly lower numbers of intracellular S.Typhimurium in CFU/ml. Data are represented as mean ±SEM. Statistical analyses were performed using a two-tailed one-sample t-test and Wilcoxon test (****p < 0.0001). **(B)** Representative graph showing % inhibition of bacterial invasion by gentamycin protection assay following a 1 hr treatment of H4 cell monolayers with *S*. Typhimurium at 37°C in 5% CO_2. Data were obtained by comparing % inhibition from CFU numbers of amylin pre-treated *Salmonella* Typhimurium to CFU numbers from untreated *Salmonella* Typhimurium. The data show a significantly higher percentage of amylin-mediated inhibition of *S*. Typhimurium invasion of H4 cells. Data are represented as mean ±SEM. Statistical analyses were done by one sample t and Wilcoxon test (****p < 0.0001). **(C)** *C. elegans* infection model wherein ingested *Candida albicans* become invasive by producing hyphae that proliferate with the worm gut, eventually killing the worms. Exposure of *C. elegans* to amylin (50µg/ml) following infection with *Candida albicans* resulted in significant rescue of amylin-treated N2 worms (n=130). Most untreated N2 worms (n=126) failed to survive the *Candida* hyphal invasion, with significant numbers dying on days 2 and 3 post-infection. **(D)** A representative graph comparing the percent survival rates of amylin-treated and vehicle-treated *C. elegans*. Statistical analyses were done using one-sample t and the Wilcoxon test (*p = 0.0177).

Supplementary Figure 5

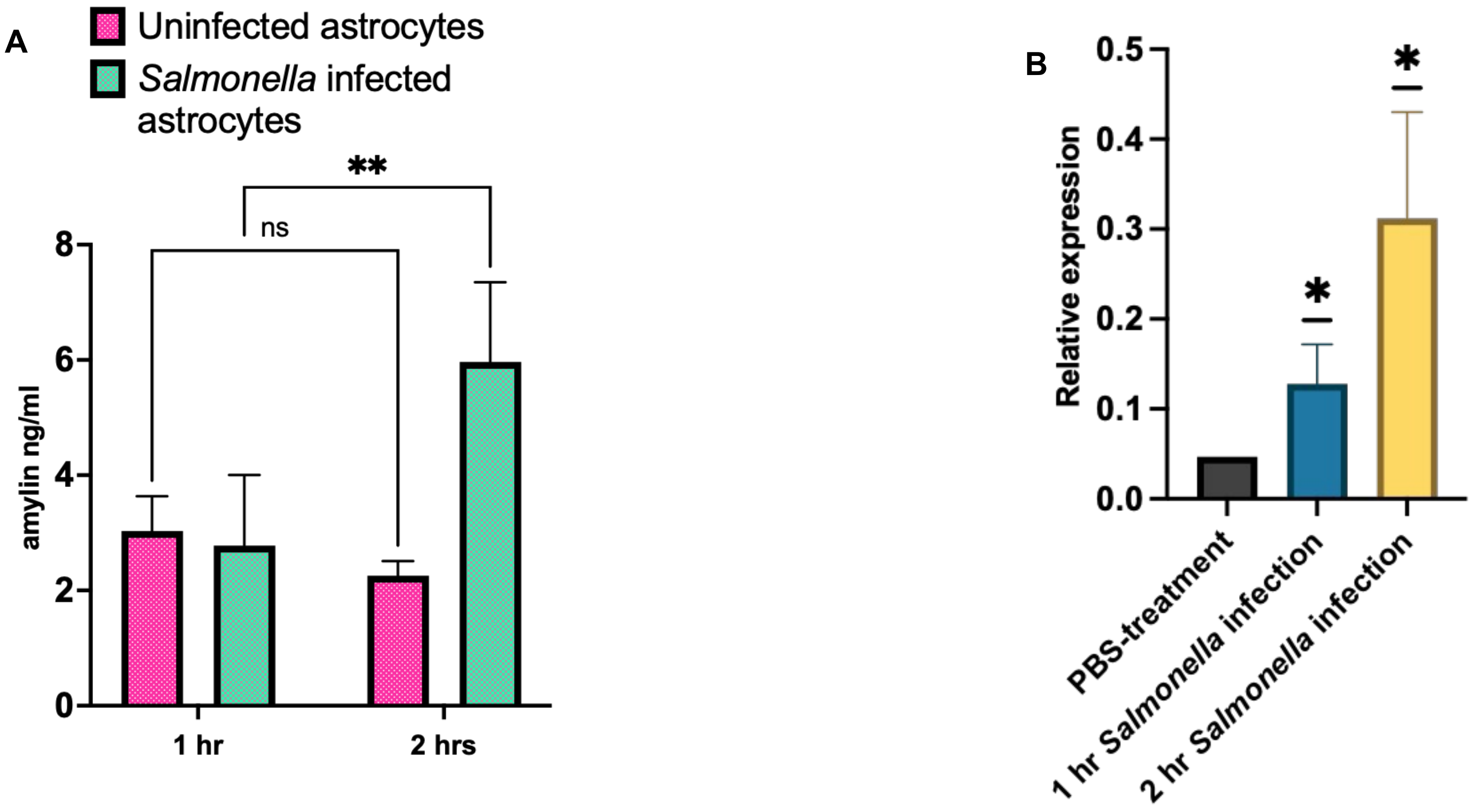

Supplementary Figure 5: Human primary astrocytes secrete amylin following infection with Salmonella *Typhimurium*. Representative figures for amylin-mediated rescue of infected human neuroglioma (H4) cells from *Salmonella* Typhimurium invasion and *C. elegans* from *Candida albicans* infection. **(A)** A representative graph shows secreted amylin from supernatants from uninfected and infected human primary astrocytes after 1 hr and 2 hrs post-S. Typhimurium infection. See also Fig. S6A. Data are represented as mean ±SEM. Statistical analyses were carried out by 2way ANOVA and Sidak's multiple comparisons test (**p = 0.0013). **(B)** A representative graph with data from an RT-PCR experiment with and without S. Typhimurium infection of astrocytes showed amylin mRNA expression levels at 1 hr and 2 hrs post-infection compared to the vehicle-treated control. See also Fig. S5B. Data are represented as mean ±SEM. Statistical analyses were conducted using one-sample t and the Wilcoxon test (*p = 0.032).

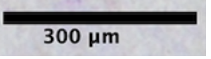

Supplementary Figure 6

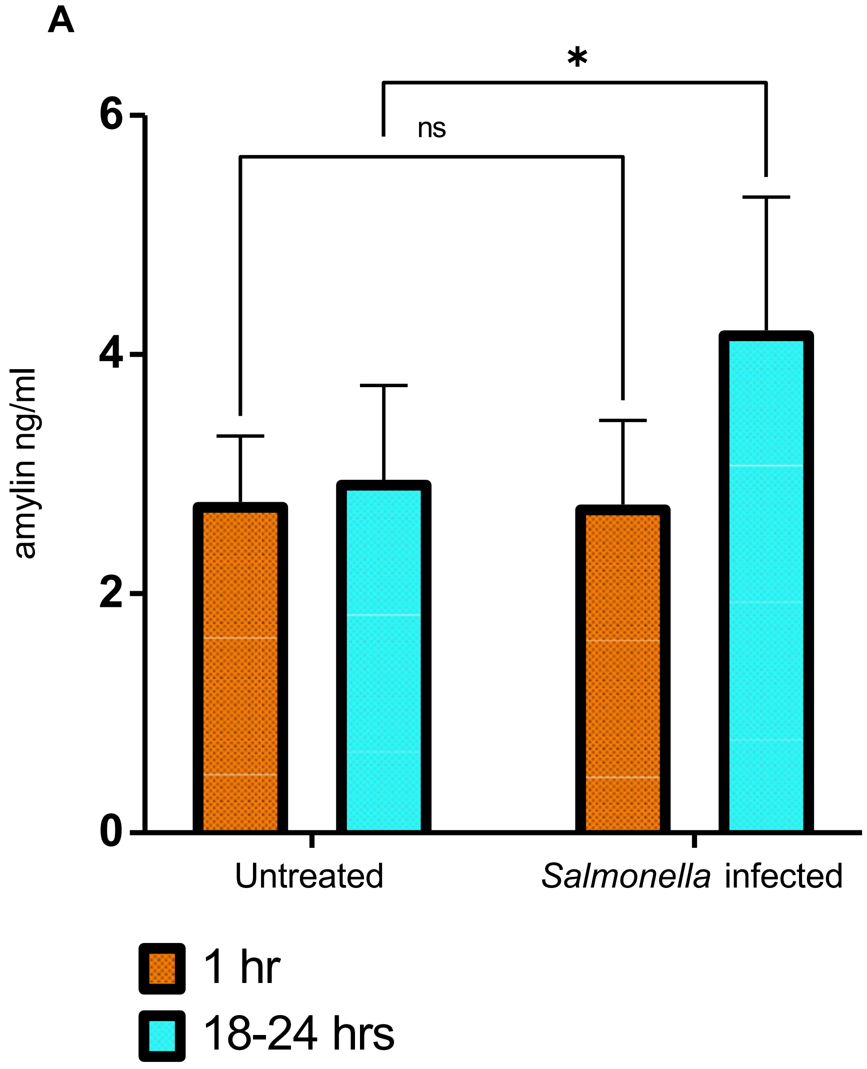

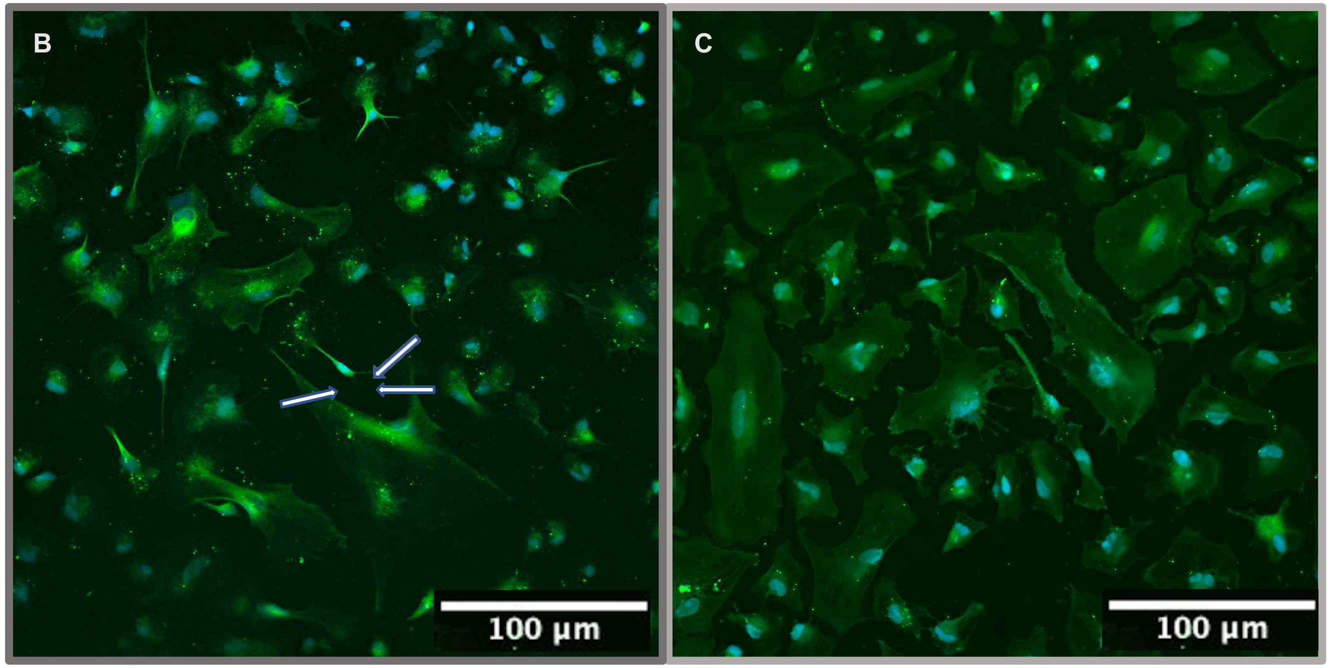

Supplementary Figure 6. Immunohistostaining of primary human astrocytes following infection with *Salmonella* Typhimurium leads to increased amylin levels versus PBS-treated astrocytes. (A) Representative graph with data from ELISA assay specific for secreted human amylin in supernatants collected from primary astrocytes cells at 1 hr and 18-24 hrs post-infection. Representative graph with data comparisons between uninfected and infected A5 and B2 cells. Data are represented as mean ±SEM. Statistical analyses were carried out by 2-way ANOVA and Sidak's multiple comparisons test ($^{*}p = 0.0409$). **(B)** Representative confocal micrograph showing anti-amylin fluorescent signal (green, Alexafluor 488) 1 hr after *Salmonella* Typhimurium infection. **(C)** Representative confocal micrograph of vehicle (PBS) – treated astrocyte. Blue represents nuclear immunostaining with DAPI.

Supplementary Figure 7

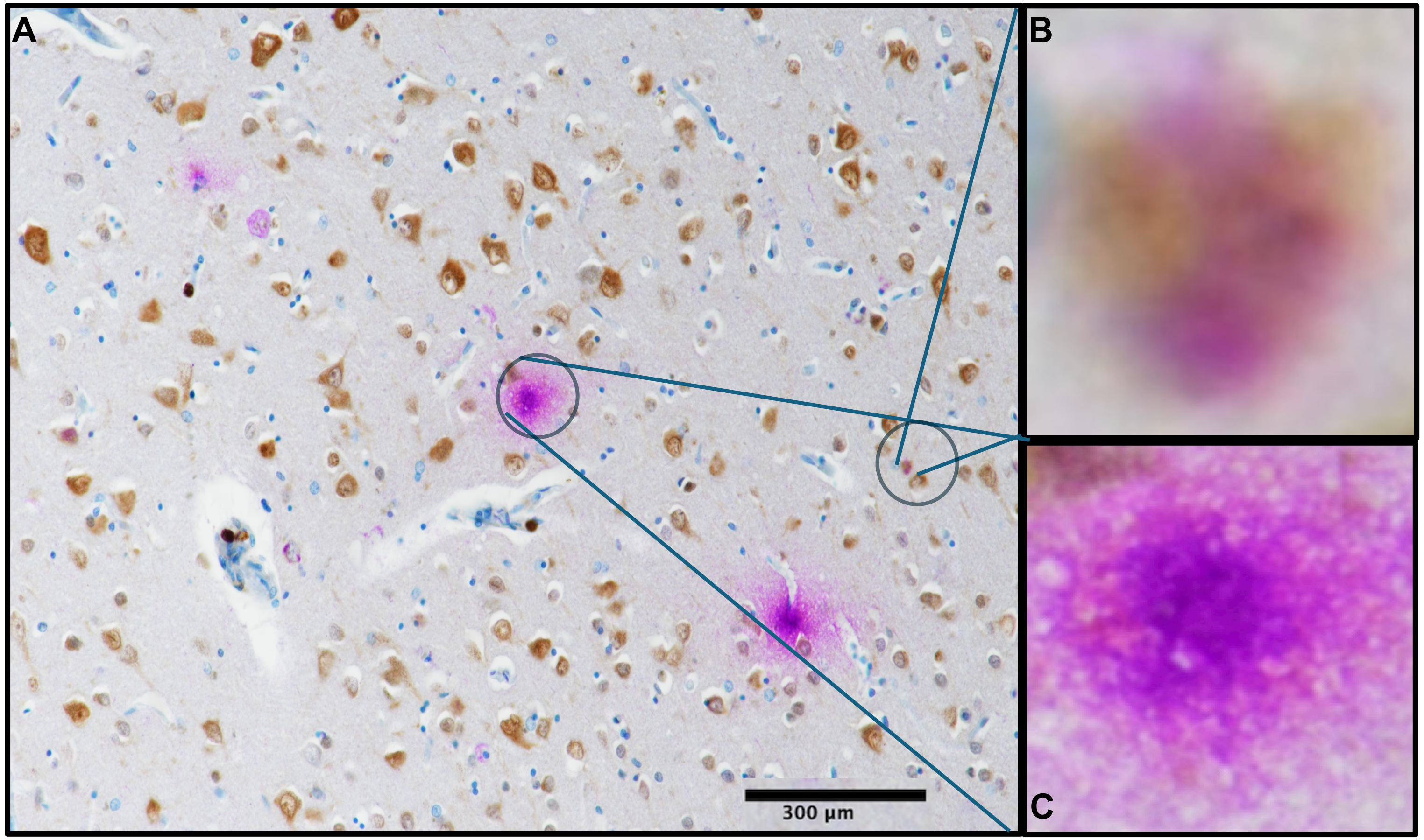

Supplementary Figure 7. Immunohistochemical Detection of Amylin, Bacteria, and Cell Nuclei in Human Brain Tissue. (A) Representative immunohistochemistry images of human brain tissue show the distribution of amylin, Gram-positive bacteria, and cell nuclei. Amylin was detected using DAB staining, appearing brown within and outside cells. Gram-positive bacteria visualized using a secondary antibody and the Ventana Purple kit, appear purple-stained. Nuclei were counterstained with hematoxylin and appear blue. **(Panels B-C)** Representative zoomed-in views highlighting amylin and Gram-positive bacterial colocalization.
